# Isolation, biochemical characterization, and genome sequencing of two high‐quality genomes of a novel chitinolytic *Jeongeupia* species

**DOI:** 10.1002/mbo3.1372

**Published:** 2023-08-06

**Authors:** Nathanael D. Arnold, Daniel Garbe, Thomas B. Brück

**Affiliations:** ^1^ Department of Chemistry Werner‐Siemens Chair for Synthetic Biotechnology (WSSB), TUM School of Natural Sciences, Technical University of Munich Garching Germany

**Keywords:** bioinformatics, comparative genomics, *Neisseria*, taxonomy

## Abstract

Chitin is the second most abundant polysaccharide worldwide as part of arthropods' exoskeletons and fungal cell walls. Low concentrations in soils and sediments indicate rapid decomposition through chitinolytic organisms in terrestrial and aquatic ecosystems. The enacting enzymes, so‐called chitinases, and their products, chitooligosaccharides, exhibit promising characteristics with applications ranging from crop protection to cosmetics, medical, textile, and wastewater industries. Exploring novel chitinolytic organisms is crucial to expand the enzymatical toolkit for biotechnological chitin utilization and to deepen our understanding of diverse catalytic mechanisms. In this study, we present two long‐read sequencing‐based genomes of highly similar *Jeongeupia* species, which have been screened, isolated, and biochemically characterized from chitin‐amended soil samples. Through metabolic characterization, whole‐genome alignments, and phylogenetic analysis, we could demonstrate how the investigated strains differ from the taxonomically closest strain *Jeongeupia naejangsanensis* BIO‐TAS4‐2^T^ (DSM 24253). In silico analysis and sequence alignment revealed a multitude of highly conserved chitinolytic enzymes in the investigated *Jeongeupia* genomes. Based on these results, we suggest that the two strains represent a novel species within the genus of *Jeongeupia*, which may be useful for environmentally friendly *N*‐acetylglucosamine production from crustacean shell or fungal biomass waste or as a crop protection agent.

## Graphical abstract

In this study, a novel chitinolytic *Jeongeupia* species “wiesaeckerbachi” was isolated from soil samples, characterized biochemically, and sequenced with the long‐read platform PacBio Sequel IIe. In silico analysis unraveled genomic differences to the closest related type strain *Jeongeupia naejangsanensis* TAS4‐2 in addition to an usually extensive chitinolytic machinery.

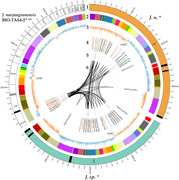

## 1 INTRODUCTION

Chitin, the second most abundant naturally occurring polysaccharide on Earth, consists of β‐(1,4)‐linked *N*‐acetyl‐d‐glucosamine and to a smaller extent, d‐glucosamine monomers. Parallelly to cellulose, which differs structurally through a lack of an amido‐functionality, and imparts stability and structure to higher plants, chitin is the principal structural component of fungal cell walls and cuticles of insects and crustacean exoskeletons, algal cell walls, and mollusks endoskeletons in aquatic organisms (Rinaudo, 2006; Younes & Rinaudo, 2015).

Despite its ubiquity, no significant long‐term accumulation could be quantified in environmental soil or sediments, implying high turnover rates by chitinolytic organisms in nature (Gooday, 1990). While glucosamine‐specific importers resemble to be widespread among bacteria (Riemann & Azam, 2002), the distribution of chitinoplastic enzymes is according to current reports limited to several groups within the phyla *Proteobacteria, Bacteroides, Actinobacteria*, and *Firmicutes* (Cottrell et al., 2000; Hunt et al., 2008). While bacteria compete with fungi for chitinous resources on land, bacteria of the orders *Vibrionales*, *Enterobacterales*, and *Neisseriales*, prevail in carbon and nitrogen cycling of polysaccharides in aquatic environments (Aumen, 1980; Beier & Bertilsson, 2013; de Boer et al., 2005; Hunt et al., 2008; Swiontek Brzezinska et al., 2014; Yu et al., 1991). Most bacterial chitinases are classified as glycoside hydrolases of family 18 (GH18) and to a vastly lesser extent, those of family 19 (Cantarel et al., 2009). Chitin composition varieties in terrestrial and aquatic environments are reflected in the formation of distinct chitinolytic systems (Bai et al., 2016).

Aquatic chitinolytic bacteria might operate with a smaller toolkit of enzymes on average (Bai et al., 2016), are not enriched on the substrate (Brzezinska et al., 2008), and exhibit generally weaker catalytic activities (Swiontek Brzezinska et al., 2014). By contrast, terrestrial bacteria are more chitinolytically active in comparison, with *Streptomyces* as the predominant genus in the early stages of chitin decomposition, whereas other *Actinomycetes* take over the reins in later stages (de Boer et al., 1999; Swiontek Brzezinska et al., 2014). Furthermore, a correlation between the abundance of bacteria and chitin decomposition rates could be observed in soil systems (Kielak et al., 2013), both of which could be promoted through the addition of substrate (Jacquiod et al., 2013; Mitchell & Alexander, 1962).

Applications of their products, the chitooligosaccharides (COS) and corresponding deacetylated derivatives comprise the food‐, cosmetic‐, wastewater treatment‐, and medical industries (Aam et al., 2010; Abu Hassan et al., 2009; Hamed et al., 2016; Rinaudo, 2006). On account of high energy costs and hazardous by‐products of chemical processes, biotechnological COS production is more sustainable and the preferred method long‐term (Beaney et al., 2005; Kaur & Dhillon, 2013; Oyeleye & Normi, 2018).

By virtue of their industrial potential and the increased relevance of sustainable (bio)technologies, extensive research on chitinases has been conducted (Binod et al., 2005; Juarez‐Jimenez et al., 2008; Lan et al., 2004; Songsiriritthigul et al., 2010; Sun et al., 2019; Vaikuntapu et al., 2016). With 10% of the global crop loss arising from plant pathogens (Strange & Scott, 2005), chitinases could gain importance as environmentally friendly crop protection agents, in particular, due to their fungal cell wall‐directed hydrolase activities (Adrangi & Faramarzi, 2013; Gomaa, 2012; Neeraja et al., 2010; Veliz et al., 2017). However, turnover rates of recalcitrant chitin represent the biggest obstacle that hinders chitinases from becoming economically feasible contenders for industrial valorization. Thus, the exploration of novel chitinolytic organisms is important to further deepen our understanding regarding catalytic mechanisms and inferred optimization of enzymes. In this respect, recent improvements regarding the costs and accessibility of next‐generation sequencing technologies enable the continuous democratization of whole‐genome sequencing. Long‐read sequencing platforms are well suited for de novo genome assembly applications, while high‐accuracy short‐read sequencing is apt for clinical variant discovery (Koboldt et al., 2013; Goodwin et al., 2016).

In this study, colloidal chitin amended soil samples were screened for chitinolytic organisms, isolated on chitin agar plates, and identified with 16S ribosomal RNA (rRNA) gene analysis (Jacquiod et al., 2013; Mitchell & Alexander, 1962). High‐fidelity genomes were created employing Pacific Biosciences' long‐read sequencer Sequel IIe and National Center for Biotechnology Information (NCBI's) Prokaryotic Genome Annotation Pipeline (PGAP). Biocomputational comparison with a highly similar Illumina NextSeq 500‐based draft genome of *Jeongeupia naejangsanensis* BIO‐TAS4‐2^T^ (Turrini et al., 2021) was utilized as a basis for taxonomic discussion. In addition, biochemical sugar metabolism capabilities were investigated utilizing API NE20 and CH50 stripes, revealing differences between the two strains investigated in this study and the type strain BIO‐TAS4‐2^T^ (Yoon et al., 2010). Finally, in silico analysis of the chitinolytic systems demonstrated the highly conserved nature within the genus *Jeongeupia* and shed light on the enzymatic composition.

## 2 MATERIALS AND METHODS

### 2.1 Chemicals and consumables

All chemicals were supplied from Sigma‐Aldrich, and general consumables were obtained from VWR. All necessary buffers and enzymes for next‐generation genome sequencing were shipped from Pacific Biosciences. High molecular weight DNA was extracted with the Quick‐DNA™ High Molecular Weight (HMW) MagBead Kit from Zymo Research and HMW genomic DNA (gDNA) shearing was conducted with g‐TUBEs (Covaris) according to the manufacturer's manual.

### 2.2 Colloidal chitin and media preparation

Colloidal chitin (CC) was prepared according to (Murthy & Bleakley, 2012) with slight modifications. Twenty grams of crab shell chitin powder (Sigma‐Aldrich) were incrementally added to 150 mL 37% HCl under moderate stirring, increasing the viscosity of the solution. When the viscosity decreased sufficiently, more chitin was carefully added. The slur was then incubated for 2–3 h at room temperature under moderate stirring, evading the formation of bubbles. Afterward, the nonviscous, fully dissolved chitin of intense brown color was slowly poured into 2 L of ice‐cold diH_2_O in a 5 L glass beaker and vigorously stirred, rapidly swelling to white colloidal chitin. The solution was incubated overnight at 4°C without stirring and neutralized the following day by adding excessive amounts of deionized water and subsequent centrifugation in a Beckman JLA8.1000 rotor for 15 min at 10,000*g* until pH 5 of the supernatant was reached. CC was harvested, autoclaved, and kept in the fridge until utilization in liquid chitinase screening media (CSM) or agar plates. The recipe was adapted and modified from (Lee et al., 1997; Singh et al., 1998): 20 g/L (2% wt/vol) CC, 0.7 g/L K_2_HPO_4_, 0.3 g/L KH_2_PO_4_, 0.5 g/L MgSO_4_ × 5 H_2_O, 10 mg/L FeSO_4_ × 7H_2_O, 20 g/L agar (optional), adjust to pH 6.5 for plates or 7 for liquid medium. After autoclaving, 0.001 g/L ZnSO_4_ and MnCl_2_ were added from sterile filtrated stock solutions before pouring of agar plates/inoculation of liquid media.

### 2.3 Soil screening and cultivation of chitinolytic organisms

**Figure 1:**
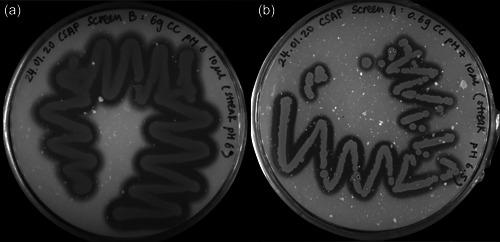
Single streaks of the chitinolytic soil bacteria strains “*J. n*.” (a) and “*J. sp*.” (b) on colloidal chitin containing (2% wt/vol) agar plates. Strains were incubated at 28°C for 3 days before documentation. Chitinase screening media of different pH values were tested, pH 6 (a) and pH 7 (b) are depicted in this figure. Enzyme activity can be deduced by translucent halos around the colony‐forming units, where chitin is degraded.

Soil samples were collected in sterile 50 mL falcon tubes and normalized to 60 g before transfer into 250 mL glass beakers. Tap water was added if the collected soil was completely dry. Afterward, samples were amended with either 1% or 10% wt/wt colloidal chitin or crab shell chitin powder (Sigma‐Aldrich) and covered with tin foil. After incubation at room temperature for 2 weeks, portions of the amended soil samples were transferred to sterile 50 mL falcon tubes and filled to 50 mL with sterile 1X PBS. Soil samples were incubated in a thermal shaker at 30°C and 600 rpm for 30 min. Supernatants were streaked out on CSM agar plates with different pH (5.5, 6, 6.5, 7, 8) using inoculation loops and incubated at 28°C for 2–3 days. Colony‐forming units (CFU) surrounded by halos were streaked onto separate CSM agar plates of the respective pH until axenic strains were obtained (Figure 1).

### 2.4 Bacterial strains

Through the method described above, the two chitinolytic bacteria *J. n*. and *J. sp*. were isolated from environmental soil samples. Species identification was realized using 16S rRNA gene analysis with the polymerase chain reaction (PCR) primer pair F27‐5′‐AGA GTT TGA TCC TGG CTC AG‐3′ and 1492R‐5′‐AAG GAG GTG ATC CAA GCC‐3′ at 55°C annealing temperature. After deploying the DreamTaq DNA polymerase (Thermo Fisher Scientific), the length and quantity of PCR products were validated via agarose gel electrophoresis, gel bands were excised, and gene fragments extracted with the Monarch DNA Gel Extraction Kit (New England BioLabs GmbH) according to the protocol. Eurofins Genomics Europe Sequencing GmbH conducted Sanger sequencing with the provided primer pair F27 and 1492R. Finally, the Geneious Prime software (v.2022.0.1) was used for read quality control, alignment, and assembly to obtain near full‐length rRNA sequences, which were compared to NCBI's 16S rRNA gene database through their BLASTn suite (Sayers et al., 2022).

### 2.5 Whole‐genome sequencing

#### 2.5.1 HMW gDNA extraction and DNA library preparation

Singular bacteria colonies were picked from CSM agar (pH 6.5) and incubated in 20 mL Tryptic Soy Broth medium in 150 mL baffled shaking flasks at 120 rpm and 28°C overnight. HMW gDNA was extracted according to the instructions of the Quick‐DNA HMW MagBead Kit (Zymo Research).

To assess the quantity and purity of the obtained DNA, 260/280 nm absorption ratios and concentrations were measured with a photometer (Nano Photometer NP80; IMPLEN) and a Qubit 4 fluorometer with the Qubit 1X dsDNA HS Assay‐Kit (Thermo Fisher Scientific). To confirm the high molarity of the gDNA, fragment sizes were analyzed with a Femto Pulse capillary electrophoresis instrument (Agilent Technologies).

When samples passed the quality control, shearing of 8 µg gDNA in 150 µL Elution Buffer was conducted with g‐TUBEs (Covaris), utilizing 1700*g* in a tabletop centrifuge. This yielded DNA fragments with a size of ca. 12 kbp, as confirmed with Femto Pulse. Subsequently, HiFi libraries were prepared according to the SMRTbell prep kit 3.0 manual, fusing barcoded adapters to the samples (Pacific Biosciences). Libraries were stored at −20°C until the day of sequencing, where primers and the polymerase bound the samples with the Sequel II Binding Kit 3.2 (Pacific Biosciences), closely following the manufacturer's recommendations.

#### 2.5.2 Sequencing

Whole genome sequencing was performed on a Sequel IIe platform (Pacific Biosciences) on a single SMRT cell (lot number 418096) with the following parameters: 2 h of pre‐extension, 2 h of adaptive loading (target p1 + p2 = 0.95) for a final on‐plate concentration of 85 pM and a 30‐h long movie window for signal detection (Ritz et al., 2023).

#### 2.5.3 Assembly and annotation

After demultiplexing with the SMRT link software (v.11.0.0.144466) to separate the barcoded reads, obtained FASTQ raw read files were assembled utilizing the Canu assembler 2.0 (Koren et al., 2017). An estimated genome size of 3.8 Mb was provided (*genomeSize* = *3.8 mb*) and the *‐pacbio* parameter was deployed; otherwise, standard settings were utilized. Log files can be found in Supporting Information: Data. Annotation was performed utilizing NCBI's PGAP (Ciufo et al., 2018; Haft et al., 2018; Li et al., 2021; Tatusova et al., 2016), which employs GeneMarkS‐2+ for gene prediction (Lomsadze et al., 2018) and TIGRFAMs for functional identification of proteins (Haft, 2001, 2003; Haft et al., 2012; Selengut et al., 2007).

#### 2.5.4 Bioinformatic analysis

Using the public Galaxy.eu server of the Galaxy web platform (Afgan et al., 2016), the following data analyses were conducted:

1. Functional genome characterization via eggNOG Mapper (Huerta‐Cepas et al., 2017; Huerta‐Cepas, Szklarczyk, et al., 2016) to retrieve Cluster of Orthologous Groups (COG) Proteins and Gene Ontology terms.
2. Genome quality assessment with CheckM (Parks et al., 2015) and BUSCO v.5.3.2 (Manni et al., 2021), based on near‐universal single‐copy orthologs. CheckM was run through Protologger, part of the Galaxy network (Hitch et al., 2021).

Visualization of the circular genome plot was realized with CIRCOS (Krzywinski et al., 2009), while the R‐Studio software with the ggplot2 package served as the main tool for the creation of all other plots, if not stated differently (Posit Team, 2022; Wickham, 2008). Mobile genetic elements and phage regions were detected with the browser‐based tool PHASTER (Arndt et al., 2016; Zhou et al., 2011). The origin of replication (ORI) was identified with DoriC 12.0 (Dong et al., 2023).

Carbohydrate active enzymes (CAZymes) were retrieved with dbCAN 3.0 (Cantarel et al., 2009; Zheng et al., 2023). Alignment and phylogenetic reconstructions of the chitin‐enacting enzymes were performed using the function “build” of ETE3 3.1.2 as implemented on the GenomeNet (Huerta‐Cepas, Serra, et al., 2016; Kyoto University Bioinformatics Center, 2023). The tree was constructed using FastTree v2.1.8 with default parameters. Values at nodes are Shimodaira–Hasegawa‐like local support (Thompson et al., 1994; Kyoto University Bioinformatics Center, 2023).

Whole genome alignment was realized with the progressiveMauve plugin within the Geneious Prime software v.2022.0.1, which is suitable for genomes containing rearranged segments due to recombination (Darling et al., 2010). Several locally collinear block (LCB) sizes were tested, whereby a compromise of conserved region count and sequence identity was selected, see Supporting Information: Data (Figure A6).

#### 2.5.5 Phylogenetic trees with Type (Strain) Genome Server (TYGS)

The genome sequence data were uploaded to the TYGS, a free bioinformatics platform available at https://tygs.dsmz.de, for a whole genome‐based taxonomic analysis (Meier‐Kolthoff & Göker, 2019). The analysis also used recently introduced methodological updates and features (Meier‐Kolthoff et al., 2022). Information on nomenclature, synonymy, and associated taxonomic literature was provided by TYGS's sister database, the List of Prokaryotic names with Standing in Nomenclature (LPSN, available at https://lpsn.dsmz.de) (Meier‐Kolthoff et al., 2022). The results were provided by the TYGS on 2023‐05‐16. The TYGS analysis was subdivided into the following steps:

1. *Determination of closest type strain genomes*: Was done in two complementary ways: First, all user genomes were compared against all type strain genomes available in the TYGS database via the MASH algorithm, a fast approximation of intergenomic relatedness (Ondov et al., 2016), and, the 10 type strains with the smallest MASH distances chosen per user genome. Second, an additional set of 10 closely related type strains was determined via the 16S rRNA gene sequences. These were extracted from the user genomes using RNAmmer (Lagesen et al., 2007). Each sequence was subsequently BLASTed (Camacho et al., 2009) against the 16S rRNA gene sequence of all 18,977‐type strains currently available in the TYGS database. This was used as a proxy to find the best 50 matching type strains (according to the bitscore) for each user genome and to subsequently calculate precise distances using the Genome BLAST Distance Phylogeny approach (GBDP) under the algorithm “coverage” and distance formula d5 (Camacho et al., 2009; Meier‐Kolthoff et al., 2013). These distances were finally used to determine the 10 closest type strain genomes for each of the user genomes.
2. *Pairwise comparison of genome sequences*: For the phylogenomic inference, all pairwise comparisons among the set of genomes were conducted using GBDP and accurate intergenomic distances inferred under the algorithm “trimming” and distance formula d5 (Meier‐Kolthoff et al., 2013). A total of 100 distance replicates were calculated each. Digital (DNA–DNA hybridization) values and confidence intervals were calculated using the recommended settings of the GGDC 3.0 (Meier‐Kolthoff et al., 2013, 2022).
3. *Phylogenetic inference*: The resulting intergenomic distances were used to infer a balanced minimum evolution tree with branch support via FASTME 2.1.6.1 including subtree‐prune‐regraft moves postprocessing (Lefort et al., 2015). Branch support was inferred from 100 pseudobootstrap replicates each. The trees were rooted at the midpoint (Farris, 1972) and visualized with PhyD3 (Kreft et al., 2017).

## 3 RESULTS AND DISCUSSION

### 3.1 Screening, isolation, and 16S rRNA gene‐based identification of chitinolytic bacteria

**Figure A1:**
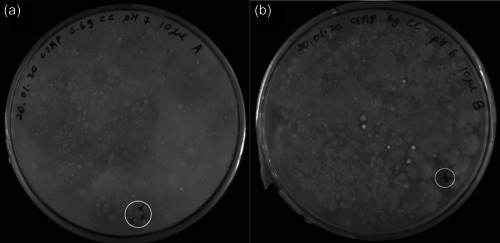
Chitinase producer screening agar plates. Chitin‐amended soil samples were incubated for 2 weeks at room temperature. The soil was moisturized with tap water if necessary. Then, sterile phosphate‐buffered saline was added, and incubated for 30 min on a thermal shaker, and resulting supernatants were streaked out on chitin agar plates with pH 6 (a) and pH 7 (b). Halos around colony‐forming units indicate chitin hydrolysis activities, due to degradation and therefore clearance of the white colloidal chitin. Colony forming units marked with an “X,” highlighted through circles, were subsequently streaked on separate chitinase producer screening agar plates.

Through the amendment of environmental soil samples with chitin in colloidal or powder form and dosages of 0.6% or 6% (wt/wt), respectively, chitinolytic microorganisms could putatively be enriched, as previously reported (Jacquiod et al., 2013). Streaking onto minimal media agar plates with 2% (wt/vol) colloidal chitin as the sole carbon‐ and nitrogen source produced CFUs, whose chitin hydrolyzing ability became visible through halos in varying diameters, indicating degradation of the paste‐like, white colloidal chitin (Figure A1). The two most promising candidates would then be subjected to 16S rRNA gene PCR (Figure 1) and identified based on BLASTn comparison with the type strain database of NCBI (Sayers et al., 2022).

Both candidates were identified as *J. naejangsanensis* strain BIO‐TAS4‐2^T^ with identical percent identities of 98.72%, query coverages of 99%, and *E* values of 0. With identities of 98.48% (*J. n*.) and 98.01% (*J. sp*.), respectively, *Jeongeupia chitinilytica*'s 16S rRNA gene showed the second most sequence homology. The transitory names were awarded based on these results, indicating that the investigated organism is *J. naejangsanensis*, leading to “*J. n*.” for the first strain. Due to visible morphological differences on the screening plates earlier in the study, possibly originating from the presence of a contaminant, the second candidate strain was thought to be a deviant *Jeongeupia*. This hypothesis was later reinforced by an aligned nucleotide identity‐based taxonomic analysis, leading to the name “*J. sp*.” When deploying the 16S rRNA gene sequences extracted from the novel high‐quality genomes (gene IDs pgaptmp_00343 [*J. n*.] and pgaptmp_1503 [*J. sp*]) to a BLASTn query, the colony 16S rRNA gene PCR‐based results could be confirmed with percent identities of 99.06%, *E* values of 0.0 and query coverages of 97%, respectively.

#### 3.1.1 Sugar metabolism

Carbon source utilization capabilities of the investigated strains were assessed using API 50CH and 20NE stripes (bioMérieux) and compared to the taxonomically closest strain *J. naejangsanensis* BIO‐TAS4.2^T^ (Table 1). As expected for closely related species, most examined characteristics were congruent, among these positive results for motility, nitrate reduction, *N‐*acetylglucosamine, d‐glucose, d‐fructose, d‐mannose, and d‐ribose. Please refer to the Supporting Information: Data for a detailed list of all results and depictions of the API stripes.

**Table 1:** Phenotypic characteristics as determined with API 20NE and API 50CH stripes from bioMérieux.

Characteristic	1	2	3
Nitrate reduction	+	+	+
*Assimilation of*			
d‐glucose	+	+	+
d‐fructose	+	+	+
d‐mannose	+	+	+
d‐ribose	+	+	+
*N*‐acetylglucosamine	+	+	+
Potassium gluconate	+	+	+
Citrate	+	+	+
Malate	+	+	+
**Capric acid**	+	−	−
**Xylitol**	+	−	−
**d‐lyxose**	+	−	−
**l‐arabitol**	+	−	−
*Hydrolysis of*			
**Esculin**	−	+	w*
**Gelatin**	−	+	+

*Note*: Taxa: 1, *J. naejangsanensi*s *BIO‐TAS4‐2(T)*, data from Yoon et al. (2010); 2, *J. n*. (this study); 3, *J. sp*. (this study). Differences between 1 and/or 2 and 3 are indicated in bold lettering. (w*) A negative result for 20NE and a weakly positive result for 50CH. See Figures A2, A3, A4 for details.

**Figure A2:**
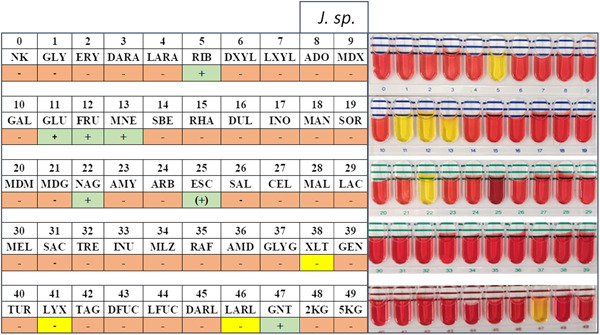
API 50CH sugar metabolism results of the chitinolytic bacterium *J. sp*. (this study). Yellow highlights on the left indicate differences to the closely related *J. naejangsanensis* BIO‐TAS4‐2 type strain (Yoon et al., 2010). Test tubes colored in red indicate an inability to utilize a given sugar, while yellow test tubes indicate a positive result based on a pH shift. Esculin in tube 25 should turn black for a positive test result. We interpreted the strong darkening as a weak positive result.

**Figure A3:**
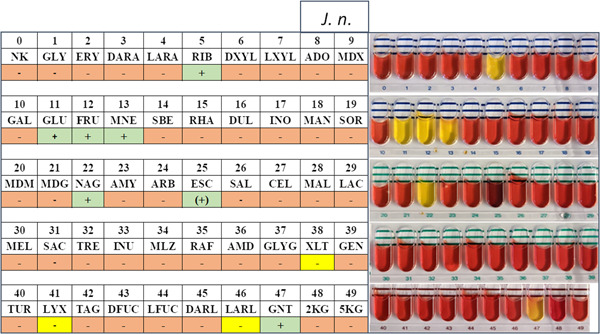
API 50CH sugar metabolism results of the chitinolytic bacterium *J. n*. (this study). Yellow highlights on the left indicate differences to the closely related *Jeongeupia naejangsanensis* BIO‐TAS4‐2 type strain (Yoon et al., 2010). Test tubes colored in red indicate an inability to utilize a given sugar, and yellow test tubes indicate a positive result based on a pH shift. Esculin in tube 25 should turn black for a positive result. We interpreted the strong darkening as a weak positive result.

**Figure A4:**
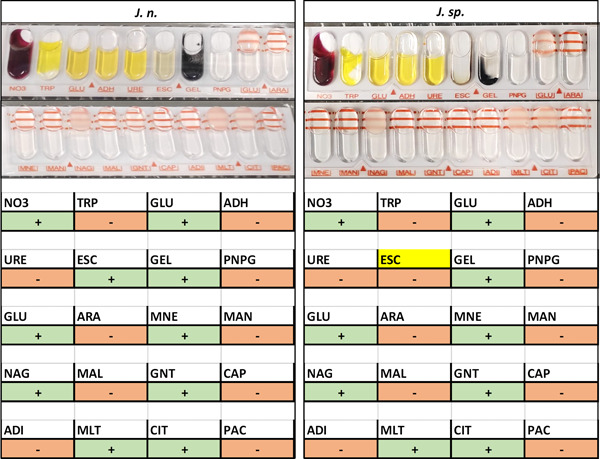
API NE 20 sugar metabolism results of the chitinolytic bacteria *J. n*. and *J. sp*. Yellow highlights indicate differences between both strains regarding their sugar metabolism. For ESCulin: A positive result is indicated through a gray/brown/black color, as seen on the left, whereas a brown coloring is visible merely at the bottom of the tube in the case of *J. sp*. ADH, l‐arginine; ADI, adipic acid; ARA, l‐arabinose; CAP, capric acid; CIT, trisoium citrate; ESC, esculin; GEL, gelatin; GLU, d‐glucose (assimilation); GLU, d‐glucose (fermentation); GNT, potassium gluconate; MAL, d‐maltose; MNE, d‐mannose; MLT, malic acid; NAG, *N*‐acetyl‐glucosamine; NO_3_, potassium nitrate; PAC, phenylacetic acid; PNPG, 4‐nitrophenyl‐βd‐galactopyranoside; TRP, l‐tryptophane; URE, urea.

Interestingly, certain differences could be illustrated regarding the assimilation of xylitol, d‐lyxose, l‐arabitol, and capric acid, all of which the type strain can utilize as a carbon source (Yoon et al., 2010). Hydrolysis of the substrates esculin and gelatine was exclusive to *J. n*. and *J. sp*. on the other hand. In this regard, a minor metabolic distinction between the two strains described in this study could be made—with *J. n*. exhibiting a more potent esculin hydrolysis capability compared to *J. sp*., as detected with the API 20NE stripe (Figure A4). These observations were mitigated by the API 50CH test results though, which demonstrated very similar β‐glucosidase activity levels based on the substrate's shading (Figures A2 and A3).

### 3.2 Genome sequencing, assembly, and quality control

Barcode adapter fused genome libraries were sequenced along other biosamples with PacBio's long‐read platform Sequel IIe (Pacific Biosciences). Subsequently, reads were demultiplexed (binned according to the barcode) computationally. The overall HiFi reads from the circular consensus sequencing mode were satisfactory in quantity and quality, with a Q36 score, translating into a 99% accurate base calling. Owed to the delicate balancing act regarding library concentrations during multiplexing, the *J. sp*. library was overrepresented, indicated by the inflated zero‐mode waveguide values and polymerase read counts compared to the *J. n*. library (Table 2). Through genome assembly with Canu 2.0 (Koren et al., 2017), reads were trimmed to 50‐ or 40‐fold remaining coverages, respectively. Full‐length circular bacterial genomes could be constructed with a length of 3.79 Mbps, while additional contigs added up to full genome sizes of 3.82/3.87 Mbps, respectively. The genomes are accessible at NCBI via the BioProject ID PRJNA978547. Obtained results are in concordance with other available genome sizes of the genus *Jeongeupia*, which range from 3.4 to 3.9 Mbps. High G + C contents of approximately 63% are also in line with 62%–65% of the other four currently known *Jeongeupia* members *J. chitinilytica* (KCTC 23701, RefSeq GCF_014652315.1), *J. naejangsanensis* (DSM 24253, RefSeq GCF_016865585.1), *J. sp*. HS‐3 (RefSeq GCF_015140455.1), and *J. sp*. USM3 (RefSeq GCF_001808185.1).

**Table 2:** Genome sequencing and assembly quality parameters.

		*J. n*.	*J. sp*.
Mean barcode quality (%)		97	98
Number of ZMWs		10,385	18,144
Polymerase reads		604,197	1,049,345
Bases		7,823,401,458	13,704,381,117
Mean read length		12,969	13,059
Coverage (fold)	Before trimming	50	200
	After trimming	50	40.14
Genome size (Mbps)		3.82	3.87
Circular contig size (Mbps)		3.79	3.79
Contigs		2	3
GC‐content (%)		63.23	63.25

Abbreviations: GC, guanine–cytosine; ZMW, zero‐mode waveguide.

**Figure A5:**
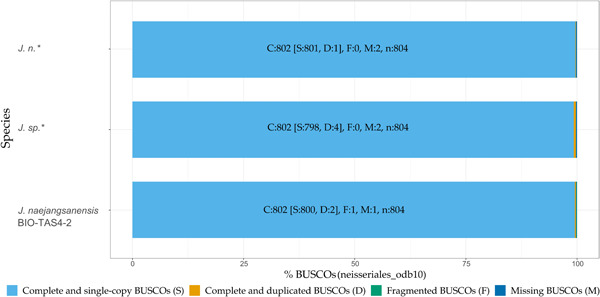
Genome quality assessment with BUSCO (v.5.3.2), based on near‐universal single‐copy orthologs in the order of *Neisseriales* (odb10). Asterisk‐labeled chitinolytic strains *J. n*. and *J. sp*. of this study were genome sequenced with a Sequel IIe platform (Pacific Biosciences). The reference genome *Jeongeupia naejangsanensis* BIO‐TAS4‐2^T^ was generated with a NextGen 500 platform (Illumina).

**Figure A6:**
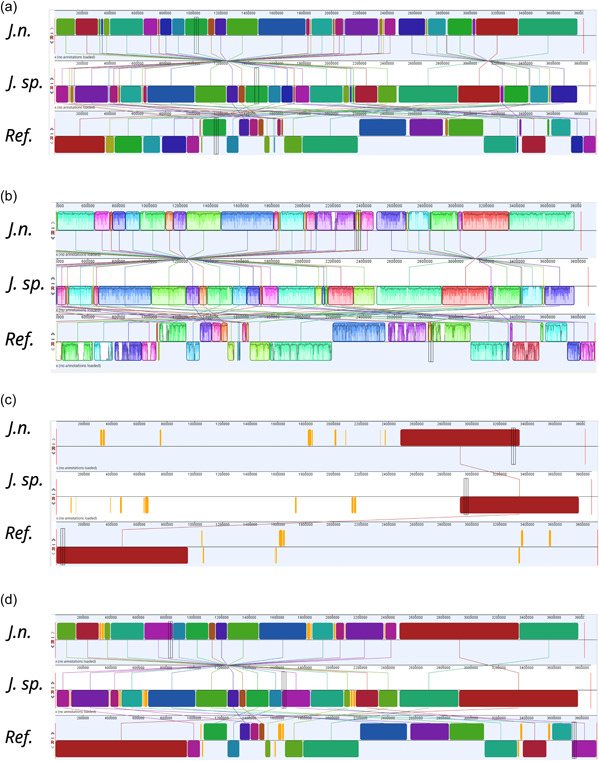
Whole‐genome sequence alignment of *J. n*., *J. sp*., and the *Jeongeupia naejangsanensis* type strain BIO‐TAS4‐2 (DSM 24253) with progressiveMauve. Locally collinear block (LCB) weights of 3,866 (a and b), 131,237 (c), and 45,296 bp (d) were applied. *J. n*. (this study) was arbitrarily set as a reference (*Ref*.). Links and identical colors indicate conserved genetic regions, low‐conserved regions are colored in orange, while nonconserved regions appear as gapA. Shifted LCBs indicate inverted regions compared to the *Ref*. at the top.

To further evaluate the assembled genome qualities, BUSCO 5.3.2 guided assessment of orthologue gene set completeness was performed, utilizing the order of the *Neisseriales* database (neisseriales_odb10) as the closest available reference (Manni et al., 2021). Calculated genome completeness was high with 99.7% and 99.8% for *J. n*. and *J. sp*., respectively (Figure A5), on par with the *J. naejangsanensis* type strain BIO‐TAS4‐2 genome (99.7%). The lack of fragmented BUSCOs as opposed to the reference strain could originate in the gapless assembly enabled by the long‐read sequencing platform. Duplicated orthologues, as prevalently seen in the *J. sp*. genome, stem exclusively from its minor secondary and tertiary contigs, which were not omitted as contaminations by the Canu 2.0 assembler.

#### 3.2.1 Genome‐based identification

*Average nucleotide identity* (*ANI)‐based identification*: The ANI values, widely accepted as a computational tool to define species boundaries and confirm identities of Bacteria and Archaea, were routinely assessed by the annotation pipeline PGAP (Ciufo et al., 2018). When *J. naejangsanensis* was chosen as the reference organism (user provided), the highest respective ANI values were 87.61% for both *J.n*. and *J. sp*. compared to *J. naejangsanensis* BIO‐TAS4‐2^T^ (RefSeq GCA_016865585.1, ASM1686558v1). In the case of submitting the genus *Jeongeupia* as a reference, the best hit changed to an aligned nucleotide identity of 85.9% for *J. sp*. with the species *J. chitinilytica* (RefSeq GCA_014652315.1, ASM1465231v1), instead (Ciufo et al., 2018). However, it remained unchanged for *J. n., still* being identified as *J. naejangsanensis* BIO‐TAS4‐2^T^. All ANIs were determined inconclusive, with values below 95%. When consulting the external fastANI and orthoANIu tools (Jain et al., 2018; Yoon et al., 2017), *J. sp*. had higher respective ANI values of 88.37% and 87.56% with *J. naejangsanensis* BIO‐TAS4‐2^T^ opposed to 86.93% and 85.5% with *J. chitinilytica*.

Possible reasons for ANI values below 95% involve genome contamination and incompleteness, the genomes belonging to a novel species, the database lacking high‐quality type strain genomes, or biogeographical effects between the strains investigated in this study (country of origin Germany) and the type strain BIO‐TAS4‐2^T^ (country of origin Korea). Furthermore, it is reported for several genera, for example, *Variovorax* or *Stenotrophomonas*, to be defined more loosely by the ANIs, with lower cutoff values of 88% and 88.5%, respectively (Ciufo et al., 2018). This might also apply to the genus of *Jeongeupia*. Genome contamination is invalidated by analysis with the CheckM tool (Parks et al., 2015), which asserts completeness of 99.57% and contamination of 1.07%/1.28% for *J. n./J. sp*., respectively. Assessment of near‐universal single‐copy orthologues through BUSCO v.5.3.2 (Manni et al., 2021) supports the notion, that genome completeness and assembly qualities were high (Figure A5).

Horizontal gene transfer and adaptation to local habitats, driven by interactions between local bacterial communities (Polz et al., 2013) should be accounted for when discussing genomic rearrangement or gene flux. Additionally, soil pH and salinity largely affect bacterial communities' composition (Fierer & Jackson, 2006; Lauber et al., 2009; Lozupone & Knight, 2007). Given the vast distance between the two sample collection sites, Naejang mountain in Korea and a billabong of the river Isar near Munich, Germany, the soil composition most likely differed. Furthermore, the distributed genome hypothesis, which states, that the gene pool of a bacterial taxon is more complex than that of an individual species, might serve to explain differences in observed genomes even within the species level, leading to genetic differences possibly reflected in ANI values (Baumdicker et al., 2012).

**Figure 2:**
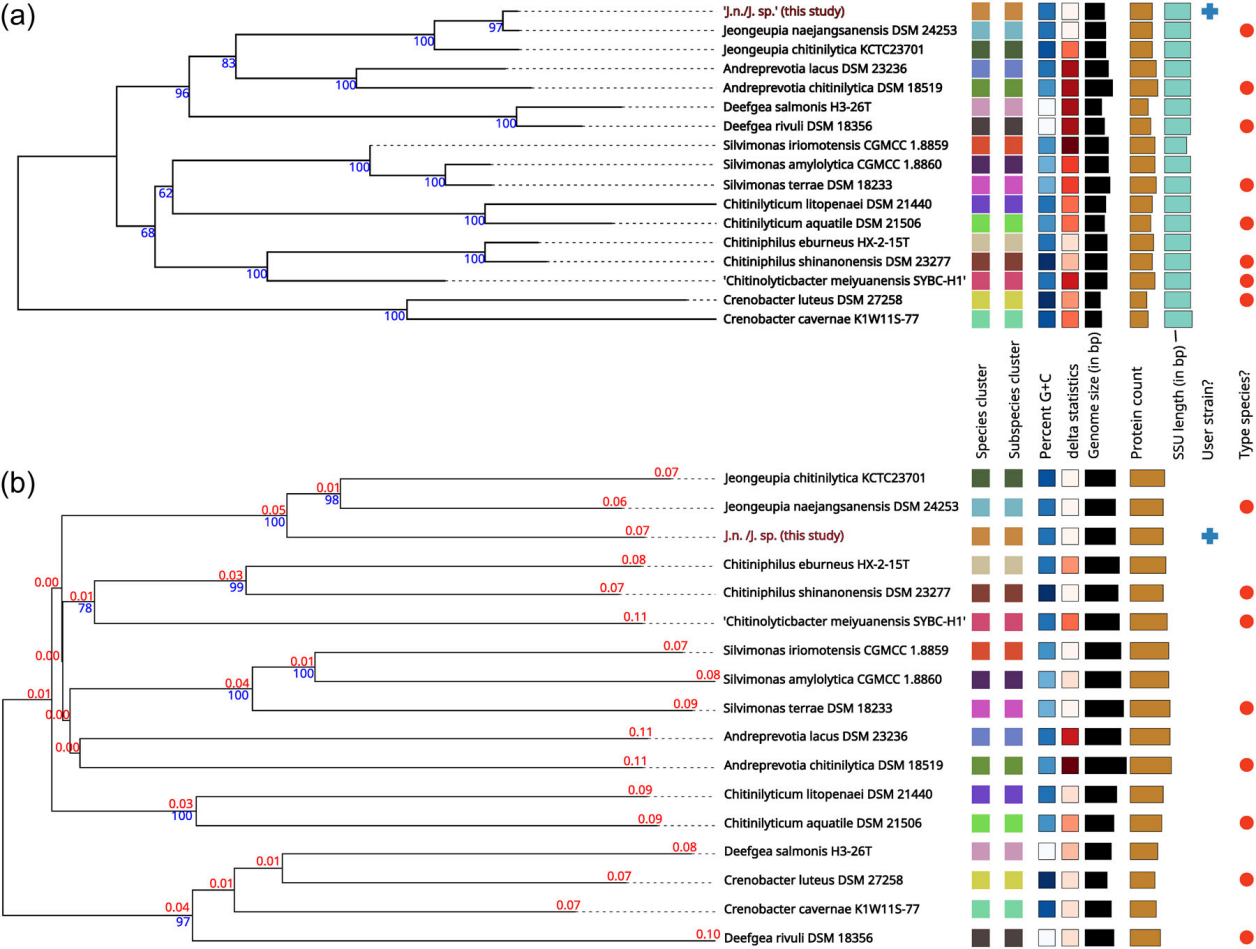
Phylogenetic trees based on 16S rRNA gene or whole‐genome sequences. Both trees were inferred with FastME 2.1.6.1 (Lefort et al., 2015) from GBDP distances calculated from 16S rRNA gene (a) or genome (b) sequences. The branch lengths are scaled in terms of the GBDP distance formula d5. The numbers above branches are GBDP pseudobootstrap support values > 60% from 100 replications, with an average branch support of 93.3% (a) or 71.2% (b). The trees were rooted at the midpoint (Farris, 1972). G + C percent values were 48.74–68.37, *δ* statistics 0.26–0.361 (a) and 0.253–0.373 (b), genome sizes (in bp) 2,854,912–5,153,521, number of proteins 2764–4454 and SSU lengths (in bp, applies for (a) only) 1285–1526. The numbers in red represent branch length values. C, cytosine; G, guanine; GBDP, Genome BLAST Distance Phylogeny; rRNA, ribosomal RNA.

*Whole‐genome sequence‐based identification*: Due to the ambiguous nature of the ANI‐based identification results, whole‐genome sequence‐based taxonomic identification was performed utilizing the free bioinformatics platform of the TYGS (Meier‐Kolthoff et al., 2022; Meier‐Kolthoff & Göker, 2019). Since the phylogenetic tree assembly incorporates a 16S rRNA gene sequence BLAST database search (Camacho et al., 2009), it is no coincidence, that our earlier results were reproduced and the investigated strains assigned closest to *J. naejangsanensis* BIO‐TAS4‐2 (DSM 24253) with a confidence value of 97% and a delta statistic of 0.26 (Holland et al., 2002) (Figure 2a). Intriguingly, when inferring the phylogenetic tree based on comparing whole‐genome sequences, the strains investigated in this study were located in their branch next to *J. naejangsanensis* and *J. chitinilytica* with a confidence value of 100% and a delta statistic of 0.258 (Figure 2b). The results described above suggest, that the bacteria *J. n*. and *J. sp*. of this study might represent identical or closely related strains of a novel species within the *Jeongeupia* genus. This hypothesis aligns with ANI values of below 95% and their closest hit, indicating diverging species in most cases, with a few taxonomic exceptions mentioned above (Ciufo et al., 2018).

#### 3.2.2 Functional annotation and chitinolytic potential

**Figure 3:**
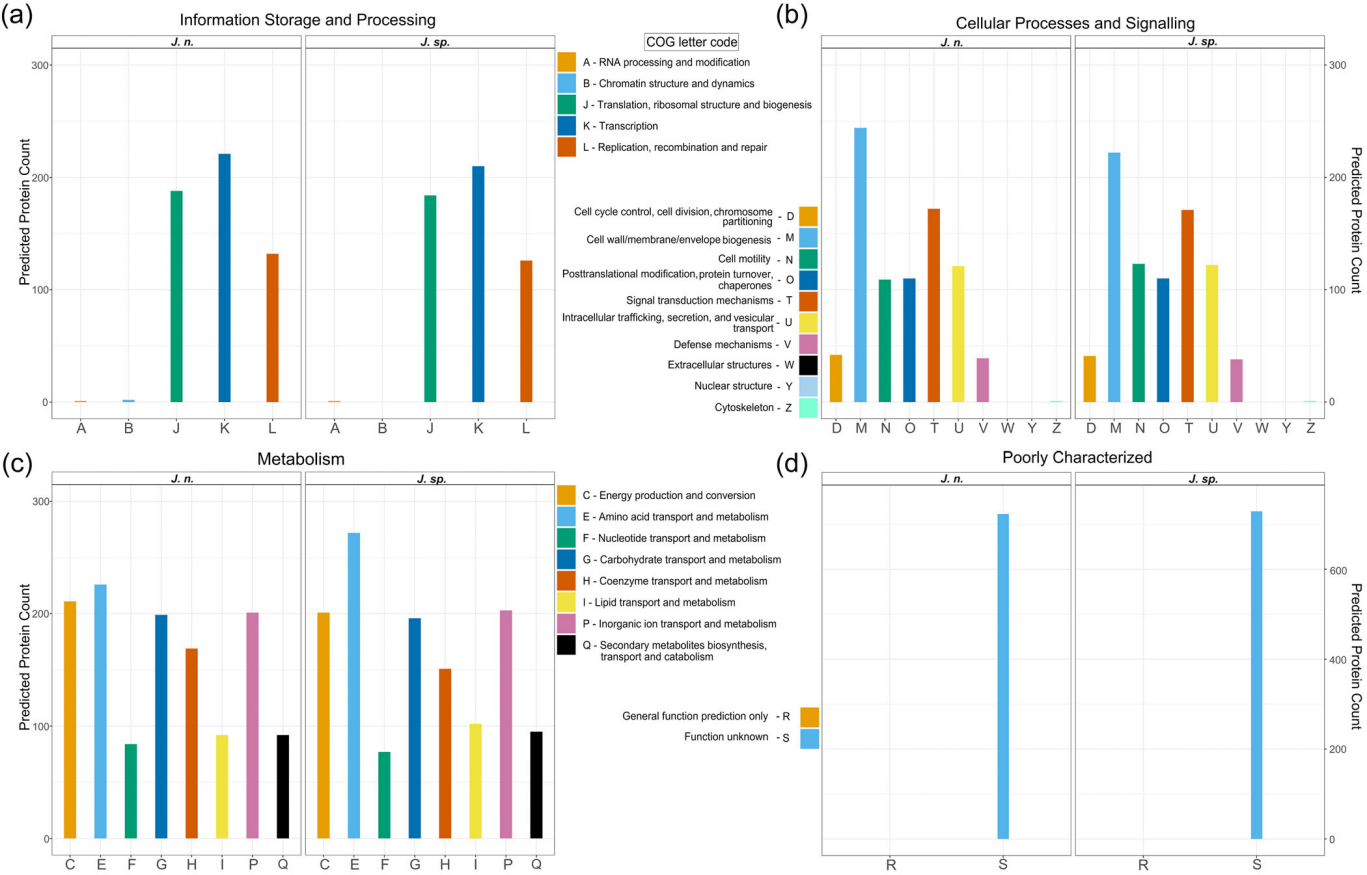
Functional annotation of the *J. n*. and *J. sp*. genomes based on Clusters of Orthologous Groups (COGs) of proteins. Please note, that the scale for the group of poorly characterized enzymes (d) differs from that of the other functional groups (a–c). For the approx. A total of 3340 unique genes each, 79% could be annotated (a–c) while 21% are of unknown function (d).

In addition to the TIGRFAM database directed annotation automatically performed by PGAP (Haft et al., 2012; Li et al., 2021), genomes were functionally categorized based on the COGs of proteins with the eggNOG‐mapper (Huerta‐Cepas et al., 2017) (Figure 3). This way, 79% of all genes could be annotated for both strains, while 21% are of unknown function, which is a typical distribution, even for genomes of the well‐studied model organism *Escherichia coli* (Cummins et al., 2022). While the two investigated genomes generally exhibit extremely similar characteristics, the results suggest, that *J. n*. possesses a higher fraction of cell wall biogenesis genes, whereas *J. sp*. has access to more amino acid transport and metabolism‐related genes. With the chitin hydrolyzing ability in mind, demonstrated both on colloidal chitin agar plates as well as in shaking flasks, the genomes were analyzed for CAZymes with dbCAN 3.0 (Zheng et al., 2023) and manually. Specifically, the chitinase (EC 3.2.1.14) containing GH18, GH19 as well as the β‐*N*‐acetyl‐hexosaminidases comprising GH20 (EC 3.2.1.52), and the central auxiliary enzyme of family 10 (AA10), the lytic polysaccharide monooxygenase (LPMO), were of interest (Drula et al., 2021; Hemsworth et al., 2014, 2015; Henrissat et al., 2023; Mekasha et al., 2017; Slámová et al., 2010). LPMOs or more specifically, lytic chitin monooxygenases (EC 1.14.99.53), are copper‐dependent oxidoreductases that can cleave recalcitrant chitin biomass (Vaaje‐Kolstad et al., 2010; Walton & Davies, 2016). Through C1 carbon atom oxidation at the glycosidic bond, fueled by O_2_ or H_2_O_2_ and a reducing agent, oligosaccharide aldonic acids are ultimately released in the process (Kuusk et al., 2018; Westereng et al., 2017). Although the majority of research focused on fungal LPMOs (AA9 and AA11), their bacterial equivalents (AA10) are reported to boost the conventional hydrolytic activity of GH18 on chitin, as well (Forsberg et al., 2016; Vaaje‐Kolstad et al., 2013). This way, 21 enzymes possibly involved in chitin degradation could be identified for *J. n*. and *J. sp*., respectively, comprising 13 GH18, 3 of which possess carbohydrate‐binding modules of family 5, 3 GH19, 3 GH20, a single β‐*N*‐acetylhexosaminidase, and a single LPMO. Based on its published annotated draft genome (GCA_016865585.1), type strain *J. naejangsanensis* BIO‐TAS4‐2 exhibited 21 potentially chitinolytic enzymes, with 13 GH18, 3 GH19, 2 GH20, a single LPMO and one, as partial chitinase annotated putative protein (Turrini et al., 2021).

According to a study from 2016, which compared the chitinolytic systems of aquatic and terrestrial chitinolytic systems based on available genomes at that time, *Jeongeupia* exhibits an exceptionally rich enzyme toolkit (Bai et al., 2016), that reminds us of fungal *Trichoderma* species (Seidl et al., 2005). To our knowledge, few bacteria, among them *Streptomyces coelicolor* A3(2) (Saito et al., 2000) and *Andreprevotia ripae* (Lorentzen et al., 2021), are described with access to comparable chitinase gene copy numbers.

**Figure 4:**
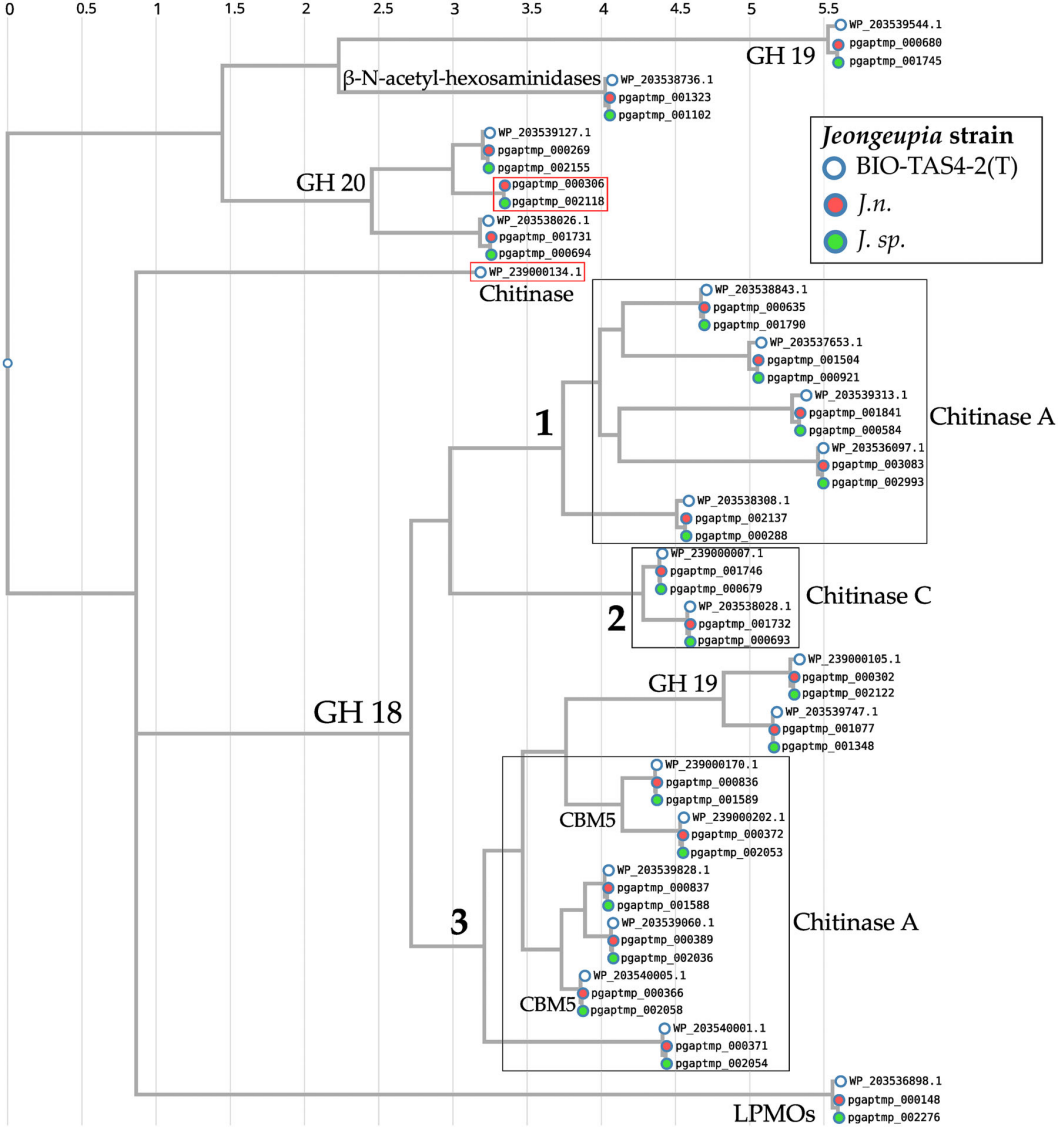
Rootless phylogenetic tree of chitin‐hydrolyzing *Jeongeupia* strains based on Shimodaira–Hasegawa‐like local support. Enzymes were data mined from the *Jeongeupia* genomes with dbCAN 3.0, clades are labeled with Clusters of Orthologous Groups and Gene Ontology terms and SWISS‐MODEL‐based functional annotation predictions. Sequence alignment was performed with CLUSTALW. The phylogenetic tree was inferred using FastTree v2.1.8 with default parameters. BIO‐TAS4‐2^T^ = *Jeongeupia naejangsanensis* reference. *J. n*. and *J. sp*. are whole‐genome sequenced strains from this study. Differences are framed in red. CBM, carbohydrate‐binding module; GH, glycoside hydrolases of family 18, 19, or 20; LPMO, lytic polysaccharide monooxygenase.

To compare the chitinolytic systems taxonomically, and reveal orthologous enzymes, a CLUSTALW sequence alignment of the translated amino sequences was performed, followed by a phylogenetic tree generation (Huerta‐Cepas, Serra, et al., 2016; Kyoto University Bioinformatics Center, 2023; Thompson et al., 1994) (Figure 4). The results suggest that the chitinolytic enzymes of the three compared *Jeongeupia* strains are highly conserved, except for one orthologous GH20 unique to the two strains of this study and one single chitinase exclusive to the type strain reference genome. Comprising the majority of bacterial chitinases, the GH18 were separated into three distinct clades, one of which could be functionally annotated as chitinase C by the eggNOG‐mapper (Huerta‐Cepas et al., 2017; Huerta‐Cepas, Szklarczyk, et al., 2016). The latter might represent the endo‐chitinases, responsible for randomized cleavage along the chitin polysaccharide chain. Despite belonging to the same family, GH18 enzymes differentiate in sequence and catalytic mechanisms (Hoell et al., 2010), which is reflected by the two separate chitinase A‐like branches, identified with the SWISS‐MODEL sequence homology database (Studer et al., 2020; Waterhouse et al., 2018). The auxiliary oxidoreductase enzyme LPMO was assigned to its own, distant branch based on sequence homology, and its oxygen‐driven mechanism, which deviates drastically from conventionally operating hydrolase‐based chitin‐active enzymes (Bissaro et al., 2017; Kuusk et al., 2018). Curiously, GH19 was represented in two separate clades, one seemingly homologous to a GH18 clade with carbohydrate‐binding module 5, while the other clade shared more sequence identity with vastly different GH20 and β‐*N*‐acetyl‐hexosaminidases.

All enzymes annotated as GH20 or β‐*N*‐acetyl‐hexosaminidases, responsible for processive exo‐chitinase activities, were assigned as descendants of a branch with three distinct clades. Since CLUSTALW is based purely on amino acid sequence alignment, the taxonomic allocation does not necessarily elucidate the singular clades' function but rather illuminates phylogenetic coherences and evolutionary processes.

#### 3.2.3 Comparison to the *J. naejangsanensis* BIO‐TAS4‐2^T^ genome

**Figure 5:**
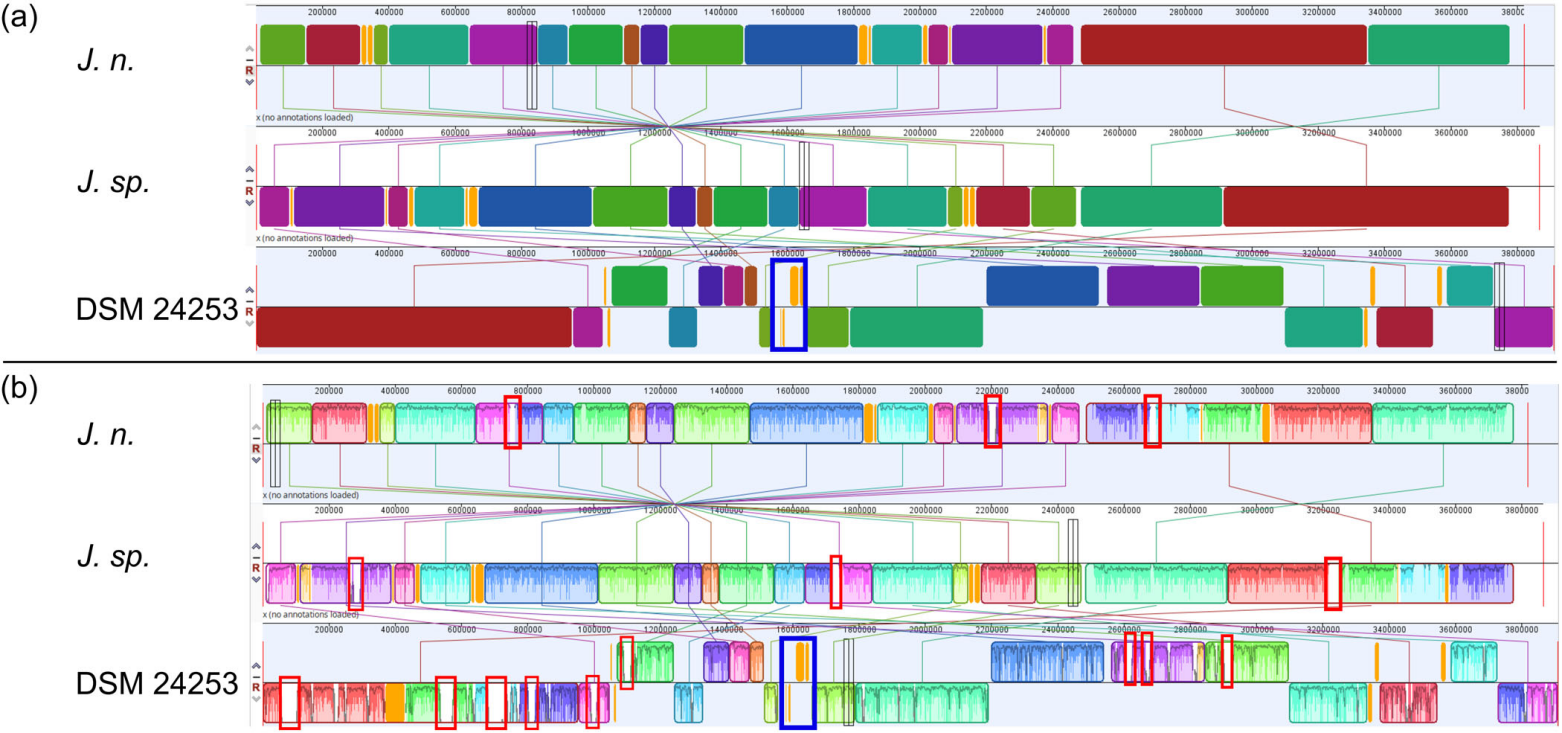
Whole‐genome sequence alignment of *J. n, J. sp*., and the *Jeongeupia naejangsanensis* type strain BIO‐TAS4‐2 (DSM 24253) with progressiveMauve. (a) A locally collinear block (LCB) weight of 45,296 bp was applied. *J. n*. (this study) was arbitrarily set as a reference. See supplementary data (Figure A6) for how different LCB weight settings affect the number of LCBs. Links and identical colors indicate conserved genetic regions, low‐conserved regions are colored in orange, while nonconserved regions appear as gaps. Shifted LCBs indicate inverted regions compared to the reference at the top. The largest coherent low/nonconserved region between BIO‐TAS4‐2^T^ and *J. n./J. sp*. is approximately 105 kb in length and framed in blue. (b) Individual sequence homologies within LCBs, where regions of low sequence identity are framed in red. Figure obtained from the progressiveMauve plugin within the Geneious Prime software (v.2022.0.1).

A whole‐genome sequence alignment was conducted with the computational tool progressiveMauve (Darling et al., 2010) (Figure 5). The software workflow includes selecting a reference sequence, followed by gapless multiple alignments of the input sequences, which serve as anchor regions. Subsequently, a phylogenetic guide tree is inferred, which is utilized to progressively apply an algorithm at every internal node, removing small matches that cause rearrangements and negatively affect the anchoring scores. Through an iterative process, progressiveMauve tries to align the sequences to maximize the conserved regions shared among the input sequences (Armstrong et al., 2019).

Figure 5 depicts, how the genomes of *J. n*. and *J. sp*. are highly conserved but entirely inverted. Orange‐shaded segments illustrate loosely conserved regions. When looking at the sequence homologies, several regions within individual LCBs, featuring low sequence identities, become apparent. These regions indicate horizontal gene transfer, where externally acquired genes interrupt otherwise conserved blocks. Furthermore, the whole‐genome sequence alignment revealed that the investigated strains share a high sequence homology with *J. naejangsanensis* BIO‐TAS4‐2^T^. However, more genetic rearrangements or inversions of singular LCBs are apparent. One prominent partially nonconserved, partially low conserved region of approximately 105,000 bp distinguishes the *Jeongeupia* genomes, indicated with a blue frame.

**Figure 6:**
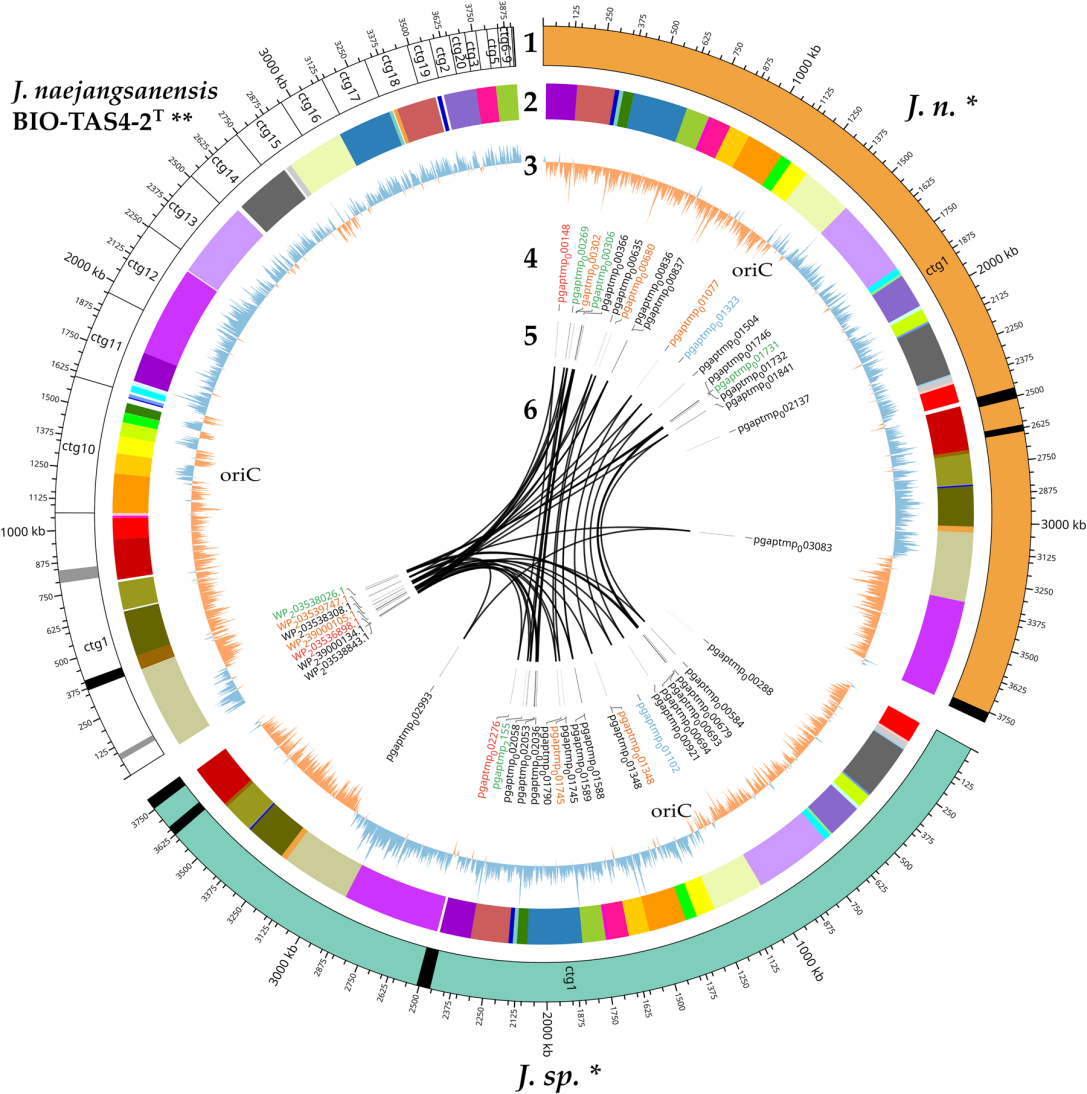
Circos plot of the two in this study generated genome sequences of *J. n*. and *J. sp*., compared to the type strain genome of *J. naejangsanensis* BIO‐TAS4‐2^T^. Circles from outermost to innermost represent (1) ideogram with contigs (ctg) and active phage regions indicated in black and inactive regions in gray, (2) conserved regions as detected with Mauve, (3) GC‐skew; regions with above average GC contents are labeled in orange, in contrast to AT richer regions labeled in blue. The origin of replication (ORI) is usually located at one of the two transition points and was identified with DoriC. (4) Chitin‐enacting enzyme CDS‐accession numbers, due to clustering, not all proteins labels could be mapped, refer to Figure 4. Red = lytic polysaccharide monooxygenase, blue = *N*‐acetyl‐hexosaminidase, black = glycosyl hydrolase family 18 (GH18, chitinase), orange = GH19, green = GH20, (5) location of respective genes, and (6) links between homologous enzymes as identified with CLUSTALW amino acid sequence alignment. Image created with CIRCOS. *, this study; **, reference.

A circular plot is a helpful tool to visualize large data amounts clearly and further highlight gaps between the presented genomes (Figure 6). Apart from the obvious advantages of a long‐read sequencer, allowing for a gapless assembly of reads into circular bacterial genomes consisting of one contig, all results presented above have been conveyed in the figure. Besides, an analysis with PHASTER (Arndt et al., 2016; Zhou et al., 2011) revealed active and inactive phage regions, which are important driving forces of gene flux and microbial evolution (Canchaya et al., 2004; Mavrich & Hatfull, 2017). *J. n*. and *J. sp*. exhibit an identical phage region pattern, which might indicate that both strains are the same organism. On the other hand, the close relative *J. naejangsanensis* BIO‐TAS4‐2^T^ has fewer phage regions overall, with more inactive regions as depicted with gray in contrast to black stripes.

Circular plotting of the chitin‐active gene loci elucidated different arrangements within the respective genomes. All chitin hydrolysis‐related genes are clustered tightly in contig 1 of the reference genome, whereas the corresponding genes are distributed more evenly in the *J. n./J. sp*. genomes, with one GH18 in a particularly remote locus. Nevertheless, both chitinase C‐like hydrolases in the genome reside in close proximity as well as two out of three GH19 and GH20 enzymes, respectively, forming small pseudo clusters.

Although the existence of distinct chitin hydrolase clusters might tempt one to assume varying enzymes, the alignment with CLUSTALW and the inferred phylogenetic tree depicted (Kyoto University Bioinformatics Center, 2023; Thompson et al., 1994) (Figure 4), that the chitinolytic enzymes are highly conserved among the *Jeongeupia* genomes, but rearranged drastically. As suggested by the progressiveMAUVE alignment, most likely through gene flux events. Overall, the three genomes differ merely in two genes: *J. naejangsanensis* BIO‐TAS4‐2^T^ has an additional chitinase WP_239000134.1 which the strains of this study lack, whereas *J. n*. and *J. sp*. have access to one additional GH20 hexosaminidase pgaptmp_000306/002118.

Lastly, GC‐skew calculation could highlight over‐ and underabundance of the nucleotides guanine and cytosine. As a result, the two eligible ORI loci per genome could be unraveled, typically placed at the transition points of nucleotide overrepresentation (Lobry, 1996). Due to the replication initiation gene *dnaA* at one of those two conversion regions, the ORI could be located exactly with the DoriC 12.0 tool (Dong et al., 2023; Kosmidis et al., 2020; Trojanowski et al., 2018). Interestingly, the *J. naejangsanensis* BIO‐TAS4‐2^T^ genome shows inconsistent regions of GC overabundance in contigs 11 and 16, which could hint at either misassembled regions or gene flux. The corresponding LCBs of *J. n*. and *J. sp*., indicated by a color code, are arranged differently and in accordance with the general GC‐skew.

## 4 CONCLUSIONS

In this study, soil sample‐derived chitinolytic organisms could be enriched through chitin amendment as demonstrated before (Jacquiod et al., 2013). Sequential screening and isolation on chitin agar plates were followed by 16S rRNA gene PCR‐guided identification, according to which the two most promising candidates were *J. naejangsanensis* BIO‐TAS4‐2^T^ (DSM 24253) (Turrini et al., 2021; Yoon et al., 2010). Long read‐sequencing with Pacific Bioscience's Sequel IIe platform and annotation with NCBI's PGAP provided high‐quality genomes of the investigated strains, as confirmed with CheckM and BUSCO (Li et al., 2021; Manni et al., 2021; Parks et al., 2015; Tatusova et al., 2016). Whole‐genome alignment revealed horizontal gene transfer and inversions of LCBs in comparison to the type strain (Darling et al., 2010; Arndt et al., 2016). Taxonomic evaluation based on aligned nucleotide identity (ANI) values yielded inconclusive results (Ciufo et al., 2018; Jain et al., 2018; Yoon et al., 2017). On the contrary, the whole‐genome alignment‐based taxonomic assessment suggested, that the two strains investigated in this study are novel *Jeongeupia* species closely related to *J. naejangsanensis* and *J. chitinilytica* (Meier‐Kolthoff & Göker, 2019). This hypothesis is further supported by the results of the biochemical characterization, which demonstrated distinct differences between the type strain BIO‐TAS4‐2^T^ and *J.n*./*J. sp*. of this study. We, therefore, propose the species name *Jeongeupia wiesaeckerbachi*, based on the name of the billabong in proximity to the organism's finding site. A thorough in silico query for enzymes involved in the chitinolytic machinery and phylogenetic analysis thereof revealed an extraordinary amount of enzymes with a high degree of conservation among the investigated *Jeongeupia* species (Thompson et al., 1994; Bai et al., 2016; Huerta‐Cepas, Serra, et al., 2016).

The novel *Jeongeupia* species presented in this study might provide a cost‐effective and environmentally friendly process to convert crustacean shell and fungal biomass waste into *N*‐acetylglucosamine based on its large set of chitin‐active enzymes. Further research must be conducted to demonstrate their suitability as antimycotic crop protection agents in a similar fashion to other studies (Neeraja et al., 2010; Swiontek Brzezinska et al., 2014). In addition, chitinases and other chitinoplastic enzymes such as chitin‐deacetylases could play significant roles in future circular bioeconomic approaches, where insects, crustaceans exoskeletons, or fungal residues are to be valorized in chemoenzymatic processes for applications in the food, chemical, cosmetic, and pharmaceutical industry (Intasian et al., 2021; Triunfo et al., 2022).

## AUTHOR CONTRIBUTIONS

**Nathanael D. Arnold**: Conceptualization (equal); data curation (equal); formal analysis (equal); investigation (equal); methodology (equal); software (equal); validation (equal); visualization (equal); writing—original draft (equal). **Daniel Garbe**: Conceptualization (equal); supervision (lead); writing—review and editing (equal). **Thomas B. Brück**: Conceptualization (equal); funding acquisition (equal); project administration (equal); resources (equal); supervision (equal); writing—review and editing (equal).

## CONFLICT OF INTEREST STATEMENT

The authors declare no conflict of interest.

## ETHICS STATEMENT

None required.

## Data Availability

All data are provided in full in the results section and the appendix of this paper. All raw datasets generated and/or analyzed during the current study are available in the Zenodo online repository: https://doi.org/10.5281/zenodo.8032359. The *J. n*. and *J. sp*. genomes can be accessed at NCBI with the BioSample IDs SAMN35557021 and SAMN35557022, respectively.

## 1

See Figures A1, A2, A3, A4, A5, A6 and Table A1.

**Table A1:** Complete API CH50 sugar metabolism results for (1) *Jeongeupia naejangsanensis* BIO‐TAS4‐2^T^ (data from (Yoon et al., 2010)), (2) *J. n*. (this study), and (3) *J. sp*. (this study).

Tube	Characteristic	1	2	3
1	Glycerol	−	**−**	**−**
2	Erythritol	**−**	**−**	**−**
3	d‐arabinose	**−**	**−**	**−**
4	l‐arabinose	**−**	**−**	**−**
5	d‐ribose	**+**	**+**	**+**
6	d‐xylose	**−**	**−**	**−**
7	l‐xylose	**−**	**−**	**−**
8	d‐xylose	**−**	**−**	**−**
9	Methyl‐beta‐d‐xylopyranoside	**−**	**−**	**−**
10	d‐galactose	**−**	**−**	**−**
11	d‐glucose	**+**	**+**	**+**
12	d‐fructose	**+**	**+**	**+**
13	d‐mannose	**+**	**+**	**+**
14	l‐sorbose	**−**	**−**	**−**
15	l‐rhamnose	**−**	**−**	**−**
16	Dulcitol	**−**	**−**	**−**
17	Inositol	**−**	**−**	**−**
18	d‐mannitol	**−**	**−**	**−**
19	d‐sorbitol	**−**	**−**	**−**
20	Methyl‐alpha‐d‐mannopyranoside	**−**	**−**	**−**
21	Methyl‐alpha‐d‐glucopyranoside	**−**	**−**	**−**
22	*N*‐acetylglucosamine	**+**	**+**	**+**
23	Amygdalin	**−**	**−**	**−**
24	Arbutin	**−**	**−**	**−**
**25**	**Esculin ferric citrate**	−	**(+)**	**(+)**
26	Salicin	**−**	**−**	**−**
27	d‐cellobiose	**−**	**−**	**−**
28	d‐maltose	**−**	**−**	**−**
29	d‐lactose (bovine origin)	**−**	**−**	**−**
30	d‐melibiose	**−**	**−**	**−**
31	d‐saccharose (sucrose)	**−**	**−**	**−**
32	d‐trehalose	**−**	**−**	**−**
33	Inulin	**−**	**−**	**−**
34	d‐melezitose	**−**	**−**	**−**
35	d‐raffinose	**−**	**−**	**−**
36	Amidon (starch)	**−**	**−**	**−**
37	Glycogen	**−**	**−**	**−**
**38**	**Xylitol**	**+**	−	**−**
39	Gentiobiose	**−**	**−**	**−**
40	d‐turanose	**−**	**−**	**−**
**41**	**d‐lyxose**	**+**	**−**	**−**
42	d‐tagatose	**−**	**−**	**−**
43	d‐fucose	**−**	**−**	**−**
44	l‐fucose	**−**	**−**	−
45	d‐arabitol	−	−	**−**
**46**	**l‐arabitol**	**+**	−	−
47	Potassium gluconate	**+**	**+**	**+**
48	Potassium 2‐ketogluconate	**−**	**−**	**−**
49	Potassium 5‐ketogluconate	**−**	**−**	**−**

*Note*: Differences are highlighted with bold type.

## References

[mbo31372-bib-0001] Aam, B. B. , Heggset, E. B. , Norberg, A. L. , Sørlie, M. , Vårum, K. M. , & Eijsink, V. G. H. (2010). Production of chitooligosaccharides and their potential applications in medicine. Marine Drugs, 8, 1482–1517. 10.3390/md8051482 20559485 PMC2885077

[mbo31372-bib-0002] Abu Hassan, M. A. , Tan, P. L. , & Zainon Noor, Z. (2009). Coagulation and flocculation treatment of wastewater in textile industry using chitosan. Journal of Chemical and Natural Resources Engineering, 4, 43–53.

[mbo31372-bib-0003] Adrangi, S. , & Faramarzi, M. A. (2013). From bacteria to human: A journey into the world of chitinases. Biotechnology Advances, 31, 1786–1795. 10.1016/j.biotechadv.2013.09.012 24095741

[mbo31372-bib-0004] Afgan, E. , Baker, D. , van den Beek, M. , Blankenberg, D. , Bouvier, D. , Čech, M. , Chilton, J. , Clements, D. , Coraor, N. , Eberhard, C. , Grüning, B. , Guerler, A. , Hillman‐Jackson, J. , Von Kuster, G. , Rasche, E. , Soranzo, N. , Turaga, N. , Taylor, J. , Nekrutenko, A. , & Goecks, J. (2016). The Galaxy platform for accessible, reproducible and collaborative biomedical analyses: 2016 update. Nucleic Acids Research, 44, W3–W10. 10.1093/nar/gkw343 27137889 PMC4987906

[mbo31372-bib-0005] Armstrong, J. , Fiddes, I. T. , Diekhans, M. , & Paten, B. (2019). Whole‐genome alignment and comparative annotation. Annual Review of Animal Biosciences, 7, 41–64. 10.1146/annurev-animal-020518-115005.Whole-Genome 30379572 PMC6450745

[mbo31372-bib-0006] Arndt, D. , Grant, J. R. , Marcu, A. , Sajed, T. , Pon, A. , Liang, Y. , & Wishart, D. S. (2016). PHASTER: A better, faster version of the PHAST phage search tool. Nucleic Acids Research, 44, W16–W21. 10.1093/nar/gkw387 27141966 PMC4987931

[mbo31372-bib-0007] Aumen, N. G. (1980). Microbial succession on a chitinous substrate in a woodland stream. Microbial Ecology, 6, 317–327. 10.1007/BF02010494 24227227

[mbo31372-bib-0008] Bai, Y. , Eijsink, V. G. H. , Kielak, A. M. , van Veen, J. A. , & de Boer, W. (2016). Genomic comparison of chitinolytic enzyme systems from terrestrial and aquatic bacteria. Environmental Microbiology, 18, 38–49. 10.1111/1462-2920.12545 24947206

[mbo31372-bib-0009] Baumdicker, F. , Hess, W. R. , & Pfaffelhuber, P. (2012). The infinitely many genes model for the distributed genome of bacteria. Genome Biology and Evolution, 4, 443–456. 10.1093/gbe/evs016 22357598 PMC3342869

[mbo31372-bib-0010] Beaney, P. , Lizardi‐Mendoza, J. , & Healy, M. (2005). Comparison of chitins produced by chemical and bioprocessing methods. Journal of Chemical Technology & Biotechnology, 80, 145–150. 10.1002/jctb.1164

[mbo31372-bib-0011] Beier, S. , & Bertilsson, S. (2013). Bacterial chitin degradation‐mechanisms and ecophysiological strategies. Frontiers in Microbiology, 4, 149. 10.3389/fmicb.2013.00149 23785358 PMC3682446

[mbo31372-bib-0012] Binod, P. , Pusztahelyi, T. , Nagy, V. , Sandhya, C. , Szakács, G. , Pócsi, I. , & Pandey, A. (2005). Production and purification of extracellular chitinases from *Penicillium aculeatum* NRRL 2129 under solid‐state fermentation. Enzyme and Microbial Technology, 36, 880–887. 10.1016/j.enzmictec.2004.12.031

[mbo31372-bib-0013] Bissaro, B. , Røhr, Å. K. , Müller, G. , Chylenski, P. , Skaugen, M. , Forsberg, Z. , Horn, S. J. , Vaaje‐Kolstad, G. , & Eijsink, V. G. H. (2017). Oxidative cleavage of polysaccharides by monocopper enzymes depends on H2O2. Nature Chemical Biology, 13, 1123–1128. 10.1038/nchembio.2470 28846668

[mbo31372-bib-0014] de Boer, W. , Folman, L. B. , Summerbell, R. C. , & Boddy, L. (2005). Living in a fungal world: impact of fungi on soil bacterial niche development. FEMS Microbiology Reviews, 29, 795–811. 10.1016/j.femsre.2004.11.005 16102603

[mbo31372-bib-0015] de Boer, W. , Gerards, S. , Klein Gunnewiek, P. J. A. , & Modderman, R. (1999). Response of the chitinolytic microbial community to chitin amendments of dune soils. Biology and Fertility of Soils, 29, 170–177. 10.1007/s003740050541

[mbo31372-bib-0016] Brzezinska, M. S. , Lalke‐Porczyk, E. , Donderski, W. , & Walczak, M. (2008). Occurrence and activity of microorganisms in shrimp waste. Current Microbiology, 57, 580–587. 10.1007/s00284-008-9246-1 18781357

[mbo31372-bib-0017] Camacho, C. , Coulouris, G. , Avagyan, V. , Ma, N. , Papadopoulos, J. , Bealer, K. , & Madden, T. L. (2009). BLAST+: Architecture and applications. BMC Bioinformatics, 10, 421. 10.1186/1471-2105-10-421 20003500 PMC2803857

[mbo31372-bib-0018] Canchaya, C. , Fournous, G. , & Brüssow, H. (2004). The impact of prophages on bacterial chromosomes. Molecular Microbiology, 53, 9–18. 10.1111/j.1365-2958.2004.04113.x 15225299

[mbo31372-bib-0019] Cantarel, B. L. , Coutinho, P. M. , Rancurel, C. , Bernard, T. , Lombard, V. , & Henrissat, B. (2009). The Carbohydrate‐Active EnZymes database (CAZy): An expert resource for glycogenomics. Nucleic Acids Research, 37, D233–D238. 10.1093/nar/gkn663 18838391 PMC2686590

[mbo31372-bib-0020] Ciufo, S. , Kannan, S. , Sharma, S. , Badretdin, A. , Clark, K. , Turner, S. , Brover, S. , Schoch, C. L. , Kimchi, A. , & DiCuccio, M. (2018). Using average nucleotide identity to improve taxonomic assignments in prokaryotic genomes at the NCBI. International Journal of Systematic and Evolutionary Microbiology, 68, 2386–2392. 10.1099/ijsem.0.002809 29792589 PMC6978984

[mbo31372-bib-0021] Cottrell, M. T. , Wood, D. N. , Yu, L. , & Kirchman, D. L. (2000). Selected chitinase genes in cultured and uncultured marine bacteria in the α‐ and γ‐subclasses of the proteobacteria. Applied and Environmental Microbiology, 66, 1195–1201. 10.1128/AEM.66.3.1195-1201.2000 10698791 PMC91962

[mbo31372-bib-0022] Cummins, E. A. , Hall, R. J. , Connor, C. , McInerney, J. O. , & McNally, A. (2022). Distinct evolutionary trajectories in the *Escherichia coli* pangenome occur within sequence types. Microbial Genomics, 8, mgen000903. 10.1099/mgen.0.000903 36748558 PMC9836092

[mbo31372-bib-0023] Darling, A. E. , Mau, B. , & Perna, N. T. (2010). Progressivemauve: Multiple genome alignment with gene gain, loss and rearrangement. PLoS One, 5, 1–17. 10.1371/journal.pone.0011147 PMC2892488 20593022

[mbo31372-bib-0024] Dong, M.‐J. , Luo, H. , & Gao, F. (2023). DoriC 12.0: An updated database of replication origins in both complete and draft prokaryotic genomes. Nucleic Acids Research, 51, D117–D120. 10.1093/nar/gkac964 36305822 PMC9825612

[mbo31372-bib-0117] Drula, E. , Garron, M.‐L. , Dogan, S. , Lombard, V. , Henrissat, B. & Henrissat, N. (2021). The carbohydrate-active enzyme database: Functions and literature. Nucleic Acids Research, 50(D1), D571–D577. 10.1093/nar/gkab1045 PMC8728194 34850161

[mbo31372-bib-0025] Farris, J. S. (1972). Estimating phylogenetic trees from distance matrices. The American Naturalist, 106, 645–668.

[mbo31372-bib-0026] Fierer, N. , & Jackson, R. B. (2006). The diversity and biogeography of soil bacterial communities. Proceedings of the National Academy of Sciences of the United States of America, 103, 626–631. 10.1073/pnas.0507535103 16407148 PMC1334650

[mbo31372-bib-0027] Forsberg, Z. , Nelson, C. E. , Dalhus, B. , Mekasha, S. , Loose, J. S. M. , Crouch, L. I. , Røhr, Å. K. , Gardner, J. G. , Eijsink, V. G. H. , & Vaaje‐Kolstad, G. (2016). Structural and functional analysis of a lytic polysaccharide monooxygenase important for efficient utilization of chitin in *Cellvibrio japonicus* . Journal of Biological Chemistry, 291, 7300–7312. 10.1074/jbc.M115.700161 26858252 PMC4817163

[mbo31372-bib-0028] Gomaa, E. Z. (2012). Chitinase production by *Bacillus thuringiensis* and *Bacillus licheniformis*: their potential in antifungal biocontrol. The Journal of Microbiology, 50, 103–111. 10.1007/s12275-012-1343-y 22367944

[mbo31372-bib-0029] Gooday, G. W. (1990). The ecology of chitin degradation. In K. C. Marshall (Ed.), Advances in microbial ecology (pp. 387–430). Springer.

[mbo31372-bib-0030] Goodwin, S. , Mcpherson, J. D. , & Mccombie, W. R. (2016). Coming of age: Ten years of next‐generation sequencing technologies. Nature Reviews Genetics, 17, 333–351. 10.1038/nrg.2016.49 PMC10373632 27184599

[mbo31372-bib-0031] Haft, D. H. (2001). TIGRFAMs: A protein family resource for the functional identification of proteins. Nucleic Acids Research, 29, 41–43. 10.1093/nar/29.1.41 11125044 PMC29844

[mbo31372-bib-0032] Haft, D. H. (2003). The TIGRFAMs database of protein families. Nucleic Acids Research, 31, 371–373. 10.1093/nar/gkg128 12520025 PMC165575

[mbo31372-bib-0033] Haft, D. H. , DiCuccio, M. , Badretdin, A. , Brover, V. , Chetvernin, V. , O'Neill, K. , Li, W. , Chitsaz, F. , Derbyshire, M. K. , Gonzales, N. R. , Gwadz, M. , Lu, F. , Marchler, G. H. , Song, J. S. , Thanki, N. , Yamashita, R. A. , Zheng, C. , Thibaud‐Nissen, F. , Geer, L. Y. , … Pruitt, K. D. (2018). RefSeq: An update on prokaryotic genome annotation and curation. Nucleic Acids Research, 46, D851–D860. 10.1093/nar/gkx1068 29112715 PMC5753331

[mbo31372-bib-0034] Haft, D. H. , Selengut, J. D. , Richter, R. A. , Harkins, D. , Basu, M. K. , & Beck, E. (2012). TIGRFAMs and genome properties in 2013. Nucleic Acids Research, 41, D387–D395. 10.1093/nar/gks1234 23197656 PMC3531188

[mbo31372-bib-0035] Hamed, I. , Özogul, F. , & Regenstein, J. M. (2016). Industrial applications of crustacean by‐products (chitin, chitosan, and chitooligosaccharides): A review. Trends in Food Science & Technology, 48, 40–50. 10.1016/j.tifs.2015.11.007

[mbo31372-bib-0036] Hemsworth, G. R. , Henrissat, B. , Davies, G. J. , & Walton, P. H. (2014). Discovery and characterization of a new family of lytic polysaccharide monooxygenases. Nature Chemical Biology, 10, 122–126. 10.1038/nchembio.1417 24362702 PMC4274766

[mbo31372-bib-0037] Hemsworth, G. R. , Johnston, E. M. , Davies, G. J. , & Walton, P. H. (2015). Lytic polysaccharide monooxygenases in biomass conversion. Trends in Biotechnology, 33, 747–761. 10.1016/j.tibtech.2015.09.006 26472212

[mbo31372-bib-0038] Henrissat, B. , Terrapon, N. , & Coutinho, P. M. (2023). Carbohydrate‐Active enZYmes. http://www.cazy.org/

[mbo31372-bib-0039] Hitch, T. C. A. , Riedel, T. , Oren, A. , Overmann, J. , Lawley, T. D. , & Clavel, T. (2021). Automated analysis of genomic sequences facilitates high‐throughput and comprehensive description of bacteria. ISME Communications, 1, 16. 10.1038/s43705-021-00017-z 36732617 PMC9723785

[mbo31372-bib-0040] Hoell, I. A. , Vaaje‐Kolstad, G. , & Eijsink, V. G. H. (2010). Structure and function of enzymes acting on chitin and chitosan. Biotechnology and Genetic Engineering Reviews, 27, 331–366. 10.1080/02648725.2010.10648156 21415904

[mbo31372-bib-0041] Holland, B. R. , Huber, K. T. , Dress, A. , & Moulton, V. (2002). δ plots: A tool for analyzing phylogenetic distance data. Molecular Biology and Evolution, 19, 2051–2059. 10.1093/oxfordjournals.molbev.a004030 12446797

[mbo31372-bib-0042] Huerta‐Cepas, J. , Forslund, K. , Coelho, L. P. , Szklarczyk, D. , Jensen, L. J. , von Mering, C. , & Bork, P. (2017). Fast Genome‐wide functional annotation through orthology assignment by eggNOG‐Mapper. Molecular Biology and Evolution, 34, 2115–2122. 10.1093/molbev/msx148 28460117 PMC5850834

[mbo31372-bib-0043] Huerta‐Cepas, J. , Serra, F. , & Bork, P. (2016). ETE 3: Reconstruction, analysis, and visualization of phylogenomic data. Molecular Biology and Evolution, 33, 1635–1638. 10.1093/molbev/msw046 26921390 PMC4868116

[mbo31372-bib-0044] Huerta‐Cepas, J. , Szklarczyk, D. , Forslund, K. , Cook, H. , Heller, D. , Walter, M. C. , Rattei, T. , Mende, D. R. , Sunagawa, S. , Kuhn, M. , Jensen, L. J. , von Mering, C. , & Bork, P. (2016). eggNOG 4.5: A hierarchical orthology framework with improved functional annotations for eukaryotic, prokaryotic and viral sequences. Nucleic Acids Research, 44, D286–D293. 10.1093/nar/gkv1248 26582926 PMC4702882

[mbo31372-bib-0045] Hunt, D. E. , Gevers, D. , Vahora, N. M. , & Polz, M. F. (2008). Conservation of the chitin utilization pathway in the vibrionaceae. Applied and Environmental Microbiology, 74, 44–51. 10.1128/AEM.01412-07 17933912 PMC2223224

[mbo31372-bib-0046] Intasian, P. , Prakinee, K. , Phintha, A. , Trisrivirat, D. , Weeranoppanant, N. , Wongnate, T. , & Chaiyen, P. (2021). Enzymes, in vivo biocatalysis, and metabolic engineering for enabling a circular economy and sustainability. Chemical Reviews, 121, 10367–10451. 10.1021/acs.chemrev.1c00121 34228428

[mbo31372-bib-0047] Jacquiod, S. , Franqueville, L. , Cécillon, S. , M. Vogel, T. , & Simonet, P. (2013). Soil bacterial community shifts after chitin enrichment: An integrative metagenomic approach. PLoS One, 8, e79699. 10.1371/journal.pone.0079699 24278158 PMC3835784

[mbo31372-bib-0048] Jain, C. , Rodriguez‐R, L. M. , Phillippy, A. M. , Konstantinidis, K. T. , & Aluru, S. (2018). High throughput ANI analysis of 90K prokaryotic genomes reveals clear species boundaries. Nature Communications, 9, 5114. 10.1038/s41467-018-07641-9 PMC6269478 30504855

[mbo31372-bib-0049] Juarez‐Jimenez, B. , Rodelas, B. , Martinez‐Toledo, M. V. , Gonzalez‐Lopez, J. , Crognale, S. , Gallo, A. M. , Pesciaroli, C. , & Fenice, M. (2008). Production of chitinolytic enzymes by a strain (BM17) of *Paenibacillus pabuli* isolated from crab shells samples collected in the east sector of central Tyrrhenian Sea. International Journal of Biological Macromolecules, 43, 27–31. 10.1016/j.ijbiomac.2007.10.022 18076982

[mbo31372-bib-0050] Kaur, S. , & Dhillon, G. S. (2013). Recent trends in biological extraction of chitin from marine shell wastes: A review. Critical Reviews in Biotechnology, 35, 44–61. 10.3109/07388551.2013.798256 24083454

[mbo31372-bib-0051] Kielak, A. M. , Cretoiu, M. S. , Semenov, A. V. , Sørensen, S. J. , & van Elsas, J. D. (2013). Bacterial chitinolytic communities respond to chitin and pH alteration in soil. Applied and Environmental Microbiology, 79, 263–272. 10.1128/AEM.02546-12 23104407 PMC3536121

[mbo31372-bib-0052] Koboldt, D. C. , Steinberg, K. M. , Larson, D. E. , Wilson, R. K. , & Mardis, E. R. (2013). The next‐generation sequencing revolution and its impact on genomics. Cell, 155, 27–38. 10.1016/j.cell.2013.09.006 24074859 PMC3969849

[mbo31372-bib-0053] Koren, S. , Walenz, B. P. , Berlin, K. , Miller, J. R. , Bergman, N. H. , & Phillippy, A. M. (2017). Canu: Scalable and accurate long‐read assembly via adaptivek‐mer weighting and repeat separation. Genome Research, 27, 722–736. 10.1101/gr.215087.116 28298431 PMC5411767

[mbo31372-bib-0054] Kosmidis, K. , Jablonski, K. P. , Muskhelishvili, G. , & Hütt, M.‐T. (2020). Chromosomal origin of replication coordinates logically distinct types of bacterial genetic regulation. NPJ Systems Biology and Applications, 6, 5. 10.1038/s41540-020-0124-1 32066730 PMC7026169

[mbo31372-bib-0055] Kreft, Ł. , Botzki, A. , Coppens, F. , Vandepoele, K. , & Van Bel, M. (2017). PhyD3: A phylogenetic tree viewer with extended phyloXML support for functional genomics data visualization. Bioinformatics, 33, 2946–2947. 10.1093/bioinformatics/btx324 28525531

[mbo31372-bib-0056] Krzywinski, M. , Schein, J. , Birol, I. , Connors, J. , Gascoyne, R. , Horsman, D. , Jones, S. J. , & Marra, M. A. (2009). Circos: An information aesthetic for comparative genomics. Genome Research, 19, 1639–1645. 10.1101/gr.092759.109 19541911 PMC2752132

[mbo31372-bib-0057] Kuusk, S. , Bissaro, B. , Kuusk, P. , Forsberg, Z. , Eijsink, V. G. H. , Sørlie, M. , & Väljamäe, P. (2018). Kinetics of H2O2‐driven degradation of chitin by a bacterial lytic polysaccharide monooxygenase. Journal of Biological Chemistry, 293, 523–531. 10.1074/jbc.M117.817593 29138240 PMC5767858

[mbo31372-bib-0058] Kyoto University Bioinformatics Center . (2023). *GenomeNet*. https://www.genome.jp/tools/ete/

[mbo31372-bib-0059] Lagesen, K. , Hallin, P. , Rødland, E. A. , Stærfeldt, H. H. , Rognes, T. , & Ussery, D. W. (2007). RNAmmer: Consistent and rapid annotation of ribosomal RNA genes. Nucleic Acids Research, 35, 3100–3108. 10.1093/nar/gkm160 17452365 PMC1888812

[mbo31372-bib-0060] Lan, X. , Ozawa, N. , Nishiwaki, N. , KODAIRA, R. , OKAZAKI, M. , & SHIMOSAKA, M. (2004). Purification, cloning, and sequence analysis of β‐N‐ acetylglucosaminidase from the chitinolytic bacterium *Aeromonas hydrophila* strain SUWA‐9. Bioscience, Biotechnology, and Biochemistry, 68, 1082–1090. 10.1271/bbb.68.1082 15170113

[mbo31372-bib-0061] Lauber, C. L. , Hamady, M. , Knight, R. , & Fierer, N. (2009). Pyrosequencing‐based assessment of soil pH as a predictor of soil bacterial community structure at the continental scale. Applied and Environmental Microbiology, 75, 5111–5120. 10.1128/AEM.00335-09 19502440 PMC2725504

[mbo31372-bib-0062] Lee, H.‐S. , Lee, H.‐J. , Choi, S.‐W. , & Her, S. (1997). Purification and characterization of antifungal chitinase from *Pseudomonas* sp. YHS‐A2. Journal of Microbiology and Biotechnology, 7, 107–113. 10.1007/s002530000408 11030578

[mbo31372-bib-0063] Lefort, V. , Desper, R. , & Gascuel, O. (2015). FastME 2.0: A comprehensive, accurate, and fast distance‐based phylogeny inference program. Molecular Biology and Evolution, 32, 2798–2800. 10.1093/molbev/msv150 26130081 PMC4576710

[mbo31372-bib-0064] Li, W. , O'Neill, K. R. , Haft, D. H. , DiCuccio, M. , Chetvernin, V. , Badretdin, A. , Coulouris, G. , Chitsaz, F. , Derbyshire, M. K. , Durkin, A. S. , Gonzales, N. R. , Gwadz, M. , Lanczycki, C. J. , Song, J. S. , Thanki, N. , Wang, J. , Yamashita, R. A. , Yang, M. , Zheng, C. , … Thibaud‐Nissen, F. (2021). RefSeq: Expanding the Prokaryotic Genome Annotation Pipeline reach with protein family model curation. Nucleic Acids Research, 49, D1020–D1028. 10.1093/nar/gkaa1105 33270901 PMC7779008

[mbo31372-bib-0065] Lobry, J. R. (1996). Asymmetric substitution patterns in the two DNA strands of bacteria. Molecular Biology and Evolution, 13, 660–665. 10.1093/oxfordjournals.molbev.a025626 8676740

[mbo31372-bib-0066] Lomsadze, A. , Gemayel, K. , Tang, S. , & Borodovsky, M. (2018). Modeling leaderless transcription and atypical genes results in more accurate gene prediction in prokaryotes. Genome Research, 28, 1079–1089. 10.1101/gr.230615.117 29773659 PMC6028130

[mbo31372-bib-0067] Lorentzen, S. B. , Arntzen, M. Ø. , Hahn, T. , Tuveng, T. R. , Sørlie, M. , Zibek, S. , Vaaje‐Kolstad, G. , & Eijsink, V. G. H. (2021). Genomic and proteomic study of andreprevotia ripae isolated from an anthill reveals an extensive repertoire of chitinolytic enzymes. Journal of Proteome Research, 20, 4041–4052. 10.1021/acs.jproteome.1c00358 34191517 PMC8802321

[mbo31372-bib-0068] Lozupone, C. A. , & Knight, R. (2007). Global patterns in bacterial diversity. Proceedings of the National Academy of Sciences of the United States of America, 104, 11436–11440. 10.1073/pnas.0611525104 17592124 PMC2040916

[mbo31372-bib-0069] Manni, M. , Berkeley, M. R. , Seppey, M. , Simão, F. A. , & Zdobnov, E. M. (2021). BUSCO update: Novel and streamlined workflows along with broader and deeper phylogenetic coverage for scoring of eukaryotic, prokaryotic, and viral genomes. Molecular Biology and Evolution, 38, 4647–4654. 10.1093/molbev/msab199 34320186 PMC8476166

[mbo31372-bib-0070] Mavrich, T. N. , & Hatfull, G. F. (2017). Bacteriophage evolution differs by host, lifestyle and genome. Nature Microbiology, 2, 17112. 10.1038/nmicrobiol.2017.112 PMC5540316 28692019

[mbo31372-bib-0071] Meier‐Kolthoff, J. P. , Auch, A. F. , Klenk, H.‐P. , & Göker, M. (2013). Genome sequence‐based species delimitation with confidence intervals and improved distance functions. BMC Bioinformatics, 14, 60. 10.1186/1471-2105-14-60 23432962 PMC3665452

[mbo31372-bib-0072] Meier‐Kolthoff, J. P. , Carbasse, J. S. , Peinado‐Olarte, R. L. , & Göker, M. (2022). TYGS and LPSN: A database tandem for fast and reliable genome‐based classification and nomenclature of prokaryotes. Nucleic Acids Research, 50, D801–D807. 10.1093/nar/gkab902 34634793 PMC8728197

[mbo31372-bib-0073] Meier‐Kolthoff, J. P. , & Göker, M. (2019). TYGS is an automated high‐throughput platform for state‐of‐the‐art genome‐based taxonomy. Nature Communications, 10, 2182. 10.1038/s41467-019-10210-3 PMC6522516 31097708

[mbo31372-bib-0074] Mekasha, S. , Byman, I. R. , Lynch, C. , & Toupalová, H. (2017). Development of enzyme cocktails for complete saccharification of chitin using mono‐component enzymes from *Serratia marcescens* . Process Biochemistry, 56, 132–138.

[mbo31372-bib-0075] Mitchell, R. , & Alexander, M. (1962). Microbiological processes associated with the use of chitin for biological control. Soil Science Society of America Journal, 26, 556–558. 10.2136/sssaj1962.03615995002600060013x

[mbo31372-bib-0076] Murthy, N. , & Bleakley, B. (2012). Simplified method of preparing colloidal chitin used for screening of chitinase‐producing microorganisms. The Internet Journal of Microbiology, 10, 1–5. 10.5580/2e93

[mbo31372-bib-0077] Neeraja, C. , Anil, K. , Purushotham, P. , Suma, K. , Sarma, P. , Moerschbacher, B. M. , & Podile, A. R. (2010). Biotechnological approaches to develop bacterial chitinases as a bioshield against fungal diseases of plants. Critical Reviews in Biotechnology, 30, 231–241. 10.3109/07388551.2010.487258 20572789

[mbo31372-bib-0078] Ondov, B. D. , Treangen, T. J. , Melsted, P. , Mallonee, A. B. , Bergman, N. H. , Koren, S. , & Phillippy, A. M. (2016). Mash: Fast genome and metagenome distance estimation using MinHash. Genome Biology, 17, 132. 10.1186/s13059-016-0997-x 27323842 PMC4915045

[mbo31372-bib-0079] Oyeleye, A. , & Normi, Y. M. (2018). Chitinase: Diversity, limitations, and trends in engineering for suitable applications. Bioscience Reports, 38, 1–21. 10.1042/BSR20180323 PMC6131217 30042170

[mbo31372-bib-0080] Parks, D. H. , Imelfort, M. , Skennerton, C. T. , Hugenholtz, P. , & Tyson, G. W. (2015). CheckM: Assessing the quality of microbial genomes recovered from isolates, single cells, and metagenomes. Genome Research, 25, 1043–1055. 10.1101/gr.186072.114.Freely 25977477 PMC4484387

[mbo31372-bib-0081] Polz, M. F. , Alm, E. J. , & Hanage, W. P. (2013). Horizontal gene transfer and the evolution of bacterial and archaeal population structure. Trends in Genetics, 29, 170–175. 10.1016/j.tig.2012.12.006.Horizontal 23332119 PMC3760709

[mbo31372-bib-0082] Posit Team . (2022). RStudio: Integrated development environment for R.

[mbo31372-bib-0083] Riemann, L. , & Azam, F. (2002). Widespread *N*‐acetyl‐d‐glucosamine uptake among pelagic marine bacteria and its ecological implications. Applied and Environmental Microbiology, 68, 5554–5562. 10.1128/AEM.68.11.5554-5562.2002 12406749 PMC129920

[mbo31372-bib-0084] Rinaudo, M. (2006). Chitin and chitosan: Properties and applications. Progress in Polymer Science, 31, 603–632. 10.1016/j.progpolymsci.2006.06.001

[mbo31372-bib-0085] Ritz, M. , Ahmad, N. , Brueck, T. , & Mehlmer, N. (2023). Comparative genome‐wide analysis of two *caryopteris* × *Clandonensis* cultivars: Insights on the biosynthesis of volatile terpenoids. Plants, 12, 632. 10.3390/plants12030632 36771729 PMC9921992

[mbo31372-bib-0086] Saito, A. , Ishizaka, M. , Francisco, P. B. , Fujii, T. , & Miyashita, K. (2000). Transcriptional co‐regulation of five chitinase genes scattered on the *Streptomyces coelicolor* A3(2) chromosome. Microbiology, 146, 2937–2946. 10.1099/00221287-146-11-2937 11065372

[mbo31372-bib-0087] Sayers, E. W. , Bolton, E. E. , Brister, J. R. , Canese, K. , Chan, J. , Comeau, D. C. , Connor, R. , Funk, K. , Kelly, C. , Kim, S. , Madej, T. , Marchler‐Bauer, A. , Lanczycki, C. , Lathrop, S. , Lu, Z. , Thibaud‐Nissen, F. , Murphy, T. , Phan, L. , Skripchenko, Y. , … Sherry, S. T. (2022). Database resources of the National Center for Biotechnology Information. Nucleic Acids Research, 50, D20–D26. 10.1093/nar/gkab1112 34850941 PMC8728269

[mbo31372-bib-0088] Seidl, V. , Huemer, B. , Seiboth, B. , & Kubicek, C. P. (2005). A complete survey of trichoderma chitinases reveals three distinct subgroups of family 18 chitinases. FEBS Journal, 272, 5923–5939. 10.1111/j.1742-4658.2005.04994.x 16279955

[mbo31372-bib-0089] Selengut, J. D. , Haft, D. H. , Davidsen, T. , Ganapathy, A. , Gwinn‐Giglio, M. , Nelson, W. C. , Richter, A. R. , & White, O. (2007). TIGRFAMs and genome properties: Tools for the assignment of molecular function and biological process in prokaryotic genomes. Nucleic Acids Research, 35, D260–D264. 10.1093/nar/gkl1043 17151080 PMC1781115

[mbo31372-bib-0090] Singh, P. P. , Shin, Y. C. , Park, C. S. , & Chung, Y. R. (1999). Biological control of fusarium wilt of cucumber by chitinolytic bacteria. Phytopathology, 89, 92–99. 10.1094/phyto.1999.89.1.92 18944809

[mbo31372-bib-0091] Slámová, K. , Bojarová, P. , Petrásková, L. , & Křen, V. (2010). Β‐*N*‐acetylhexosaminidase: What's in a name? Biotechnology Advances, 28, 682–693. 10.1016/j.biotechadv.2010.04.004 20438826

[mbo31372-bib-0092] Songsiriritthigul, C. , Lapboonrueng, S. , Pechsrichuang, P. , Pesatcha, P. , & Yamabhai, M. (2010). Expression and characterization of *Bacillus licheniformis* chitinase (ChiA), suitable for bioconversion of chitin waste. Bioresource Technology, 101, 4096–4103. 10.1016/j.biortech.2010.01.036 20133129

[mbo31372-bib-0093] Strange, R. N. , & Scott, P. R. (2005). Plant disease: A threat to global food security. Annual Review of Phytopathology, 43, 83–116. 10.1146/annurev.phyto.43.113004.133839 16078878

[mbo31372-bib-0094] Studer, G. , Rempfer, C. , Waterhouse, A. M. , Gumienny, R. , Haas, J. , & Schwede, T. (2020). QMEANDisCo‐distance constraints applied on model quality estimation. Bioinformatics, 36, 1765–1771. 10.1093/bioinformatics/btz828 31697312 PMC7075525

[mbo31372-bib-0095] Sun, X. , Li, Y. , Tian, Z. , Qian, Y. , Zhang, H. , & Wang, L. (2019). A novel thermostable chitinolytic machinery of *Streptomyces* sp. F‐3 consisting of chitinases with different action modes. Biotechnology for Biofuels, 12, 136. 10.1186/s13068-019-1472-1 31171937 PMC6545677

[mbo31372-bib-0096] Swiontek Brzezinska, M. , Jankiewicz, U. , Burkowska, A. , & Walczak, M. (2014). Chitinolytic microorganisms and their possible application in environmental protection. Current Microbiology, 68, 71–81. 10.1007/s00284-013-0440-4 23989799 PMC3889922

[mbo31372-bib-0097] Tatusova, T. , DiCuccio, M. , Badretdin, A. , Chetvernin, V. , Nawrocki, E. P. , Zaslavsky, L. , Lomsadze, A. , Pruitt, K. D. , Borodovsky, M. , & Ostell, J. (2016). NCBI Prokaryotic Genome Annotation Pipeline. Nucleic Acids Research, 44, 6614–6624. 10.1093/nar/gkw569 27342282 PMC5001611

[mbo31372-bib-0098] Thompson, J. D. , Higgins, D. G. , & Gibson, T. J. (1994). CLUSTAL W: Improving the sensitivity of progressive multiple sequence alignment through sequence weighting, position‐specific gap penalties and weight matrix choice. Nucleic Acids Research, 22, 4673–4680. 10.1093/nar/22.22.4673 7984417 PMC308517

[mbo31372-bib-0099] Triunfo, M. , Tafi, E. , Guarnieri, A. , Salvia, R. , Scieuzo, C. , Hahn, T. , Zibek, S. , Gagliardini, A. , Panariello, L. , Coltelli, M. B. , De Bonis, A. , & Falabella, P. (2022). Characterization of chitin and chitosan derived from *Hermetia illucens*, a further step in a circular economy process. Scientific Reports, 12, 6613. 10.1038/s41598-022-10423-5 35459772 PMC9033872

[mbo31372-bib-0100] Trojanowski, D. , Hołówka, J. , & Zakrzewska‐Czerwińska, J. (2018). Where and when bacterial chromosome replication starts: A single cell perspective. Frontiers in Microbiology, 9, 2819. 30534115 10.3389/fmicb.2018.02819 PMC6275241

[mbo31372-bib-0101] Turrini, P. , Artuso, I. , Lugli, G. A. , Frangipani, E. , Ventura, M. , & Visca, P. (2021). Draft genome sequence and polyhydroxyalkanoate biosynthetic potential of *Jeongeupia naejangsanensis* type strain DSM 24253. Microbiol Resour Announc, 10, e00167‐21. 10.1128/mra.00167-21 33858927 PMC8050969

[mbo31372-bib-0102] Vaaje‐Kolstad, G. , Horn, S. J. , Sørlie, M. , & Eijsink, V. G. H. (2013). The chitinolytic machinery of *Serratia marcescens*—A model system for enzymatic degradation of recalcitrant polysaccharides. FEBS Journal, 280, 3028–3049. 10.1111/febs.12181 23398882

[mbo31372-bib-0103] Vaaje‐Kolstad, G. , Westereng, B. , Horn, S. J. , Liu, Z. , Zhai, H. , Sørlie, M. , & Eijsink, V. G. H. (2010). An oxidative enzyme boosting the enzymatic conversion of recalcitrant polysaccharides. Science, 330, 219–222. 10.1126/science.1192231 20929773

[mbo31372-bib-0104] Vaikuntapu, P. R. , Rambabu, S. , Madhuprakash, J. , & Podile, A. R. (2016). A new chitinase‐D from a plant growth promoting *Serratia marcescens* GPS5 for enzymatic conversion of chitin. Bioresource Technology, 220, 200–207. 10.1016/j.biortech.2016.08.055 27567481

[mbo31372-bib-0105] Veliz, E. A. , Martínez‐Hidalgo, P. , & M. Hirsch, A. (2017). Chitinase‐producing bacteria and their role in biocontrol. AIMS Microbiology, 3, 689–705. 10.3934/microbiol.2017.3.689 31294182 PMC6604996

[mbo31372-bib-0106] Walton, P. H. , & Davies, G. J. (2016). On the catalytic mechanisms of lytic polysaccharide monooxygenases. Current Opinion in Chemical Biology, 31, 195–207. 10.1016/j.cbpa.2016.04.001 27094791

[mbo31372-bib-0107] Waterhouse, A. , Bertoni, M. , Bienert, S. , Studer, G. , Tauriello, G. , Gumienny, R. , Heer, F. T. , de Beer, T. A. P. , Rempfer, C. , Bordoli, L. , Lepore, R. , & Schwede, T. (2018). SWISS‐MODEL: Homology modelling of protein structures and complexes. Nucleic Acids Research, 46, W296–W303. 10.1093/nar/gky427 29788355 PMC6030848

[mbo31372-bib-0108] Westereng, B. , Arntzen, M. Ø. , Agger, J. W. , Vaaje‐Kolstad G. , & Eijsink V. G. H. , (2017). Analyzing activities of lytic polysaccharide monooxygenases by liquid chromatography and mass spectrometry. Methods in Molecular Biology, 1588, 71–92. 10.1007/978-1-4939-6899-2_7 28417362

[mbo31372-bib-0109] Wickham, H. (2008). Elegant graphics for data analysis: ggplot2. Springer.

[mbo31372-bib-0110] Yoon, J. H. , Choi, J. H. , Kang, S. J. , Choi, N. S. , Lee, J. S. , & Song, J. J. (2010). *Jeongeupia naejangsanensis* gen. nov., sp. nov., a cellulose‐degrading bacterium isolated from forest soil from Naejang Mountain in Korea. International Journal of Systematic and Evolutionary Microbiology, 60, 615–619. 10.1099/ijs.0.012591-0 19654346

[mbo31372-bib-0111] Yoon, S. H. , Ha, S. , Lim, J. , Kwon, S. , & Chun, J. (2017). A large‐scale evaluation of algorithms to calculate average nucleotide identity. Antonie Van Leeuwenhoek, 110, 1281–1286. 10.1007/s10482-017-0844-4 28204908

[mbo31372-bib-0112] Younes, I. , & Rinaudo, M. (2015). Chitin and chitosan preparation from marine sources. Structure, properties and applications. Marine Drugs, 13, 1133–1174. 10.3390/md13031133 25738328 PMC4377977

[mbo31372-bib-0113] Yu, C. , Lee, A. M. , Bassler, B. L. , & Roseman, S. (1991). Chitin utilization by marine bacteria. A physiological function for bacterial adhesion to immobilized carbohydrates. Journal of Biological Chemistry, 266, 24260–24267. 10.1016/S0021-9258(18)54223-X 1761531

[mbo31372-bib-0114] Zheng, J. , Ge, Q. , Yan, Y. , Zhang, X. , Huang, L. , & Yin, Y. (2023). dbCAN3: Automated carbohydrate‐active enzyme and substrate annotation. Nucleic Acids Research, 51, W115–W121. 10.1093/nar/gkad328 37125649 PMC10320055

[mbo31372-bib-0115] Zhou, Y. , Liang, Y. , Lynch, K. H. , Dennis, J. J. , & Wishart, D. S. (2011). PHAST: A fast phage search tool. Nucleic Acids Research, 39, W347–W352. 10.1093/nar/gkr485 21672955 PMC3125810

